# Percutaneous Electrolysis and Microelectrolysis for Rotator Cuff Tendinopathy: A Systematic Review and Meta-Analysis of Randomized Controlled Trials

**DOI:** 10.3390/jcm15207983

**Published:** 2026-10-15

**Authors:** Hernán Andrés de la Barra Ortiz, Claudio Chamorro Lange, Óscar Ronzio

**Affiliations:** 1 Exercise and Rehabilitation Sciences Institute, School of Physical Therapy, Faculty of Rehabilitation Sciences, Universidad Andres Bello, Santiago 7591538, Chile; claudio.chamorro@unab.cl; 2 Department of Physical Therapy–Kinesiology, Universidad Maimónides, Buenos Aires C1405BCK, Argentina; oronzio@gmail.com

**Keywords:** musculoskeletal disorders, rotator cuff tendinopathy, shoulder pain, percutaneous electrolysis, microelectrolysis, systematic review

## Abstract

**Background/Objectives**: Rotator cuff tendinopathy frequently contributes to shoulder pain and functional limitation. This systematic review and meta-analysis examined the effects of percutaneous electrolysis (EL) and microelectrolysis (MEL) on pain, shoulder-related disability, range of motion (ROM), and pressure pain threshold (PPT), and summarized treatment-related adverse events. **Methods**: Randomized controlled trials (RCTs) were identified through searches of Scopus, PubMed, CINAHL, Web of Science, the Cochrane Library, MEDLINE, Google Scholar, ScienceDirect, and the Physiotherapy Evidence Database (PEDro), from database inception to 14 September 2026. Methodological quality, risk of bias, and certainty of evidence were evaluated using the PEDro scale, Cochrane Risk of Bias 2 tool, and GRADE framework, respectively. Random-effects models were used to pool standardized mean differences (SMDs) or mean differences (MDs) with 95% confidence intervals (CIs). **Results**: Eight RCTs involving 330 participants were included. The overall post-treatment effect favored electrolysis-based interventions for pain intensity, with a large effect size (SMD = −1.44; 95% CI: −2.21 to −0.67). Pain improvements persisted at the first follow-up within the first 3 months (MD = −2.25; 95% CI: −3.40 to −1.09) and at 6 months (MD = −2.89; 95% CI: −3.68 to −2.10). Shoulder-related disability also favored electrolysis-based interventions (SMD = −0.81; 95% CI: −1.25 to −0.36). Improvements were also observed for selected ROM outcomes, whereas evidence for PPT remained inconclusive. Adverse-event reporting varied across trials; reported events were predominantly transient and non-serious, and no serious treatment-related adverse events were reported. Overall certainty of evidence ranged from low to moderate. **Conclusions**: Electrolysis-based interventions may improve pain intensity and shoulder-related disability and may provide benefits for selected ROM outcomes in adults with rotator cuff tendinopathy. However, confidence in the pain findings remains limited, with low-certainty evidence for the primary post-treatment analysis and low-to-moderate certainty at follow-up. No serious treatment-related adverse events were reported, although the available RCT evidence was insufficient to estimate adverse-event incidence precisely. Adequately powered RCTs directly comparing EL and MEL are needed to clarify their relative clinical effectiveness.

## 1. Introduction

Shoulder pain represents a highly prevalent musculoskeletal problem worldwide and is commonly encountered in primary care settings [1]. It contributes considerably to functional disability, healthcare use, and absence from work. Rotator cuff (RC) disorders account for approximately one-third of shoulder pain presentations and are a leading cause of upper-extremity pain and disability [1,2]. In the general population, RC tendinopathy has been reported to occur at an annual incidence of approximately 0.3–5.5%, with prevalence estimates ranging from 0.5% to 7.4% [2,3,4]. Although structural RC abnormalities become more prevalent with age and are frequently observed in asymptomatic individuals, indicating a limited association between imaging findings and clinical symptoms [1,5], RC tendinopathy remains clinically relevant because of its impact on pain, function, quality of life, and participation in daily, occupational, and sporting activities [1,2].

RC tendinopathy is now recognized as a multifactorial disorder involving biological, mechanical, metabolic, and psychosocial mechanisms [1,6,7]. It is characterized by impaired tendon remodeling and a poor correlation between structural abnormalities and clinical symptoms, supporting its recognition as a clinical pain disorder rather than solely a structural tendon lesion [1,5,8,9].

Current clinical practice guidelines recommend individualized conservative management of RC tendinopathy centered on exercise therapy, education, load management, and shared decision-making, with adjunctive interventions considered according to the clinical presentation [1,8,9]. However, clinical outcomes remain variable, and many individuals continue to experience persistent pain and disability, supporting the investigation of adjunctive interventions to optimize rehabilitation outcomes [3,8].

Percutaneous electrolysis has emerged as a minimally invasive adjunct to rehabilitation for tendinopathies and other musculoskeletal disorders [10,11,12,13,14]. Both conventional percutaneous electrolysis (EL) and microelectrolysis (MEL) involve the percutaneous insertion of an acupuncture-like needle electrode into the intended target tissue, usually under ultrasound guidance, followed by the application of direct current [10,11,12,13,14]. In RC tendinopathy, the target tissue may include the affected tendon or myofascial trigger points within rotator cuff muscles, depending on the treatment protocol [10,15,16]. The proposed mechanisms underlying percutaneous electrolysis remain hypothetical and are thought to involve the mechanical stimulus of needle insertion together with the electrochemical effects of direct current. These mechanisms have been proposed to induce localized electrochemical changes within the target tissue that may contribute to pain modulation and influence inflammatory and reparative processes potentially involved in tendon healing [10,12,13,15]. EL is typically delivered in the milliampere range (approximately 1–6 mA), whereas MEL uses substantially lower intensities (100–990 μA; 0.10–0.99 mA) [10,12,16,17]. Although both approaches share a similar therapeutic rationale, they differ in current intensity, current density, application time, total electrical charge, and potentially patient tolerance [10,12]. These differences may contribute to the variability in treatment protocols and clinical responses reported across studies.

Evidence from recent systematic reviews indicates that EL and MEL may contribute to pain reduction and improved disability outcomes when used alongside exercise-based or other conservative rehabilitation approaches [10,13,14,15,17]. However, the certainty of the evidence remains low to very low because of the limited number of randomized controlled trials (RCTs) and substantial clinical and methodological heterogeneity across tendon conditions, intervention protocols, comparators, and follow-up periods [9,12,13,15,16]. Moreover, previous reviews have generally synthesized evidence across different tendinopathies, while recently published RCTs in RC tendinopathy warrant an updated condition-specific synthesis focused on both EL and MEL [10,14,15,17]. Therefore, this systematic review and meta-analysis aimed to evaluate the effectiveness of EL and MEL for pain intensity and shoulder-related disability, as well as other clinically relevant outcomes, including range of motion (ROM) and pressure pain threshold (PPT), and to summarize the adverse events reported in adults with RC tendinopathy.

## 2. Materials and Methods

### 2.1. Study Design

This systematic review and meta-analysis followed the Preferred Reporting Items for Systematic Reviews and Meta-Analyses (PRISMA 2020) recommendations and was prospectively registered with the International Prospective Register of Systematic Reviews (PROSPERO; CRD420261427531; registration date: 18 June 2026) [18,19]. The PRISMA checklist has been included as Supplementary Material File A rather than as Supplementary Material and has been cited accordingly in the manuscript.

### 2.2. Eligibility Criteria

Study eligibility was defined using the PICOS framework, covering Population, Intervention, Comparator, Outcomes, and Study Design [20]. Eligible studies were RCTs, including parallel-group and crossover designs, enrolling adults (≥18 years) with symptomatic RC tendinopathy, defined by a clinical presentation consistent with symptomatic involvement of one or more RC tendons based on shoulder symptoms and physical examination findings in accordance with current clinical practice guidelines [1]. Ultrasonography, magnetic resonance imaging, or both were considered supportive diagnostic information when reported [1,8]. Studies involving participants diagnosed with subacromial impingement syndrome (SAIS), subacromial pain syndrome (SAPS), or shoulder impingement syndrome were also eligible when the reported clinical presentation and diagnostic criteria were compatible with symptomatic RC tendon involvement [1,9]. Eligible interventions included EL and MEL, administered either alone or as components of conservative rehabilitation programs, including therapeutic exercise, manual therapy, electrophysical agents, or multimodal rehabilitation. No restrictions were applied regarding electrolysis modality, current intensity, treatment duration, treatment frequency, number of sessions, ultrasound guidance, needle characteristics, or total electrical dosage. When a shared background rehabilitation program was provided, this was recorded for both groups. Some trials evaluated the incremental effect of adding EL or MEL to an otherwise identical rehabilitation program, whereas others compared electrolysis-based multimodal interventions with active comparator programs containing different co-interventions. These comparison structures were considered when grouping studies and interpreting pooled effects. Comparisons in which EL or MEL was evaluated against no treatment were analyzed separately. Eligible comparators included sham or placebo electrolysis, therapeutic exercise, manual therapy, electrophysical agents, multimodal rehabilitation, pharmacological treatment, usual care, other conservative rehabilitation interventions, or no intervention.

The primary outcomes were pain intensity and shoulder-related disability, assessed using validated outcome measures, including but not limited to the Visual Analog Scale (VAS), Numeric Pain Rating Scale (NPRS), Shoulder Pain and Disability Index (SPADI), and Disabilities of the Arm, Shoulder and Hand (DASH) questionnaire [21,22,23]. Secondary outcomes, when reported, included PPT, ROM, muscle strength, tendon morphology, electromyographic variables, and adverse events.

Studies were excluded when shoulder pain was attributable to conditions other than RC tendinopathy, including full-thickness RC tears; RC tear arthropathy; acute traumatic shoulder disorders (e.g., fractures or glenohumeral instability); adhesive capsulitis; inflammatory, infectious, or neoplastic shoulder conditions; previous shoulder surgery; recent corticosteroid injections; systemic rheumatologic diseases; neurological disorders affecting the cervical spine or upper limb; or other non-tendinous sources of shoulder pain. Studies published in English, Spanish, or Portuguese were eligible for inclusion. No restrictions were applied regarding publication date.

### 2.3. Search Strategy

Electronic searches covered Scopus, MEDLINE, PubMed, Web of Science, ScienceDirect, the Cochrane Library, the Physiotherapy Evidence Database (PEDro), and the Cumulative Index to Nursing and Allied Health Literature (CINAHL), from database inception through 14 September 2026. Google Scholar was searched separately as an additional source for study identification. These sources were chosen to ensure broad coverage and high retrieval sensitivity for the systematic review [24]. The search strategy combined controlled vocabulary, including Medical Subject Headings (MeSH) terms, when available, and free-text terms related to the intervention, including “Electrolysis”, “Electroacupuncture”, “Direct Current”, “Intratissue Percutaneous Electrolysis”, and “Microelectrolysis”, and the target condition, including “Musculoskeletal Pain”, “Tendinopathy”, “Rotator Cuff”, “Shoulder Pain”, and “Shoulder Impingement Syndrome”, using the Boolean operators AND and OR. No language filters were applied during the electronic searches, and no restrictions regarding publication date or publication status were applied. The complete search strategy for each database is provided in Supplementary Table S1. Reference lists of included studies and relevant reviews were also manually screened for additional eligible studies.

### 2.4. Selection Process and Data Extraction

All records identified through the search strategy were imported into the Rayyan web application (Rayyan Systems Inc., Cambridge, MA, USA) to facilitate duplicate removal and independent study screening [25]. Titles and abstracts were first screened independently by two reviewers (HDB and CCH), who subsequently assessed potentially eligible reports in full text against the predefined criteria. Records published in languages other than English, Spanish, or Portuguese were screened at the title and abstract stage using the English-indexed titles, abstracts, and bibliographic information available in the databases. None of these records met the clinical and methodological criteria warranting full-text assessment; therefore, no Chinese- or Korean-language full-text report required translation for eligibility assessment or data extraction. Any disagreement was discussed and resolved with the involvement of a third reviewer (OR).

Data extraction was independently performed by the same reviewers using a standardized Microsoft Excel spreadsheet developed a priori. Extracted data included bibliographic and participant characteristics, diagnostic criteria, intervention and comparator characteristics, electrolysis treatment parameters, outcome measures, assessment time points, adverse events, and funding sources. For quantitative outcomes, group-specific sample sizes, means, and standard deviations (SDs) were extracted at each relevant assessment time point. When available, between-group effect estimates and 95% confidence intervals (CIs) were also extracted; otherwise, they were calculated from the available group-level data using the same effect measure applied in the corresponding synthesis. A summary of the study-level data used in the quantitative and narrative syntheses is provided in Supplementary Table S2. Corresponding authors were contacted when additional information or clarification was required.

### 2.5. Methodological Quality and Risk of Bias

The methodological quality of the included RCTs was assessed using the Physiotherapy Evidence Database (PEDro) scale, a 10-point methodological quality scale [26]. PEDro scores were obtained from the PEDro database whenever available; otherwise, studies were independently assessed by two reviewers (HDB and CCH). Scores were classified as excellent (9–10), good (6–8), fair (4–5), or poor (<4) according to Cashin and McAuley [26]. Disagreements were resolved by consensus with a third reviewer (OR) [20].

Three reviewers (HDB, CCH, and OR) independently evaluated risk of bias using the Cochrane Risk of Bias 2 (RoB 2) tool. The assessment covered five domains: the randomization process, deviations from intended interventions, missing outcome data, outcome measurement, and selection of the reported result [27]. Domain-level and overall judgments were categorized as low risk, some concerns, or high risk in accordance with the RoB 2 algorithm [27]. Agreement among reviewers was estimated using Fleiss’ kappa [28]. Any differences were resolved through discussion and consensus, and risk-of-bias figures were produced using the Robvis tool [29].

### 2.6. Assessment of Certainty of Evidence

The certainty of the evidence for each outcome was assessed using the Grading of Recommendations Assessment, Development and Evaluation (GRADE) approach [30,31]. Certainty was evaluated across the domains of RoB, inconsistency, indirectness, imprecision, and publication bias and rated as high, moderate, low, or very low [30]. Comparison-specific GRADE assessments were presented only when supported by at least two RCTs, whereas overall pooled effects were graded for each meta-analyzed efficacy outcome. Single-study comparisons were not graded separately but contributed to the overall pooled estimates when applicable. Pain intensity and shoulder-related disability were predefined as critical outcomes for clinical decision-making, whereas pressure pain threshold, shoulder range of motion (ROM), muscle strength, and other secondary outcomes were considered important outcomes [30,31]. The Summary of Findings table was generated using GRADEpro GDT (McMaster University and Evidence Prime Inc., Hamilton, ON, Canada).

### 2.7. Statistical Analysis

Studies were pooled in a meta-analysis when at least two were clinically and methodologically comparable and reported sufficient data for the same outcome and comparison. When exact follow-up times differed across studies, the first available follow-up within the first 3 months after treatment was considered the early follow-up assessment. Separate meta-analyses were conducted according to electrolysis modality (EL or MEL), comparator, outcome, and follow-up duration whenever sufficient data were available. Given their shared therapeutic rationale, an overall pooled estimate across both modalities was also calculated when studies were considered sufficiently comparable, while modality-specific analyses were retained to preserve the distinction between EL and MEL.

Continuous outcomes were pooled using mean differences (MDs) when the same instrument was used across studies or standardized mean differences (SMDs) when different validated instruments assessed the same outcome. Hedges’ g was used to account for small-sample bias [32,33]. Continuous outcomes were synthesized using the inverse-variance method. Adverse-event data were summarized descriptively and were not pooled quantitatively. Studies explicitly reporting adverse events were distinguished from those reporting no adverse events or providing insufficient information regarding adverse-event ascertainment. Absence of adverse-event reporting was not interpreted as evidence of zero events. The magnitude of pooled SMDs was interpreted as small (0.15), moderate (0.45), or large (0.80) according to Swinton et al. [33]. When outcomes were pooled as MDs, clinical relevance was interpreted, where available, using published minimal clinically important differences (MCIDs) for the corresponding outcome measures. Interpretation focused on the magnitude and precision of the effect estimates, together with their clinical relevance where applicable, rather than on statistical significance alone.

When SDs were unavailable but standard errors (SEs) were reported, SDs were calculated according to Cochrane recommendations [20]. When continuous data were presented as medians with interquartile ranges (IQRs), they were converted to means and standard deviations (SDs) following the approach of Wan et al. [34]. If numerical values were reported only in figures, they were retrieved with WebPlotDigitizer software (version 4.6; Pacifica, CA, USA) [35]. For multi-arm trials, shared intervention or comparator groups were handled according to Cochrane recommendations to avoid double-counting participants while preserving all relevant comparisons [20]. For crossover RCTs, paired analyses accounting for within-participant correlation were planned. If substantial carry-over was considered likely, based on the characteristics of the intervention and the adequacy of the washout period, first-period data would have been used when available [20]. However, all included trials used parallel-group designs; therefore, no crossover-specific adjustments were required.

A random-effects model was prespecified because clinical and methodological heterogeneity across studies was anticipated. Random-effects meta-analyses of continuous outcomes were performed using the restricted maximum likelihood (REML) estimator with Knapp–Hartung adjustment. Between-study heterogeneity was assessed using Cochran’s Q test and quantified by τ^2^ and the I^2^ statistic. Following Cochrane guidance, heterogeneity was interpreted as low for I^2^ values of 0–40%, moderate for 30–60%, substantial for 50–90%, and considerable for 75–100% [20].

Predefined subgroup analyses were conducted according to electrolysis modality (EL or MEL), comparator, and follow-up duration whenever sufficient data were available. Risk-of-bias sensitivity analyses were performed for critical meta-analyzed outcomes by excluding studies judged to have a high overall RoB, when applicable. When quantitative synthesis was not feasible because of substantial clinical or methodological heterogeneity or insufficient data, findings were summarized narratively. Potential small-study effects were explored through visual inspection of funnel plots and Egger’s regression test when at least 10 studies were available for a given meta-analysis [20,36]. Statistical analyses were performed using R (version 4.4.1; R Foundation for Statistical Computing, Vienna, Austria), and statistical significance was set at *p* < 0.05. Where reported, *p*-values were considered alongside effect estimates and 95% CIs and were not used as the sole basis for interpreting the findings.

## 3. Results

### 3.1. Search Results

Database searches retrieved 3029 records from PubMed, Scopus, Web of Science, CINAHL, MEDLINE, ScienceDirect, the Cochrane Library, and PEDro, while Google Scholar contributed an additional 151 records (search updated 14 September 2026) [24]. After 2290 duplicate records were removed, 739 records underwent title and abstract screening. Of these, 724 were excluded, and 15 reports were subsequently evaluated in full text. Eight RCTs fulfilled the eligibility criteria and were included in the qualitative and quantitative syntheses [37,38,39,40,41,42,43,44].

**Figure 1:**
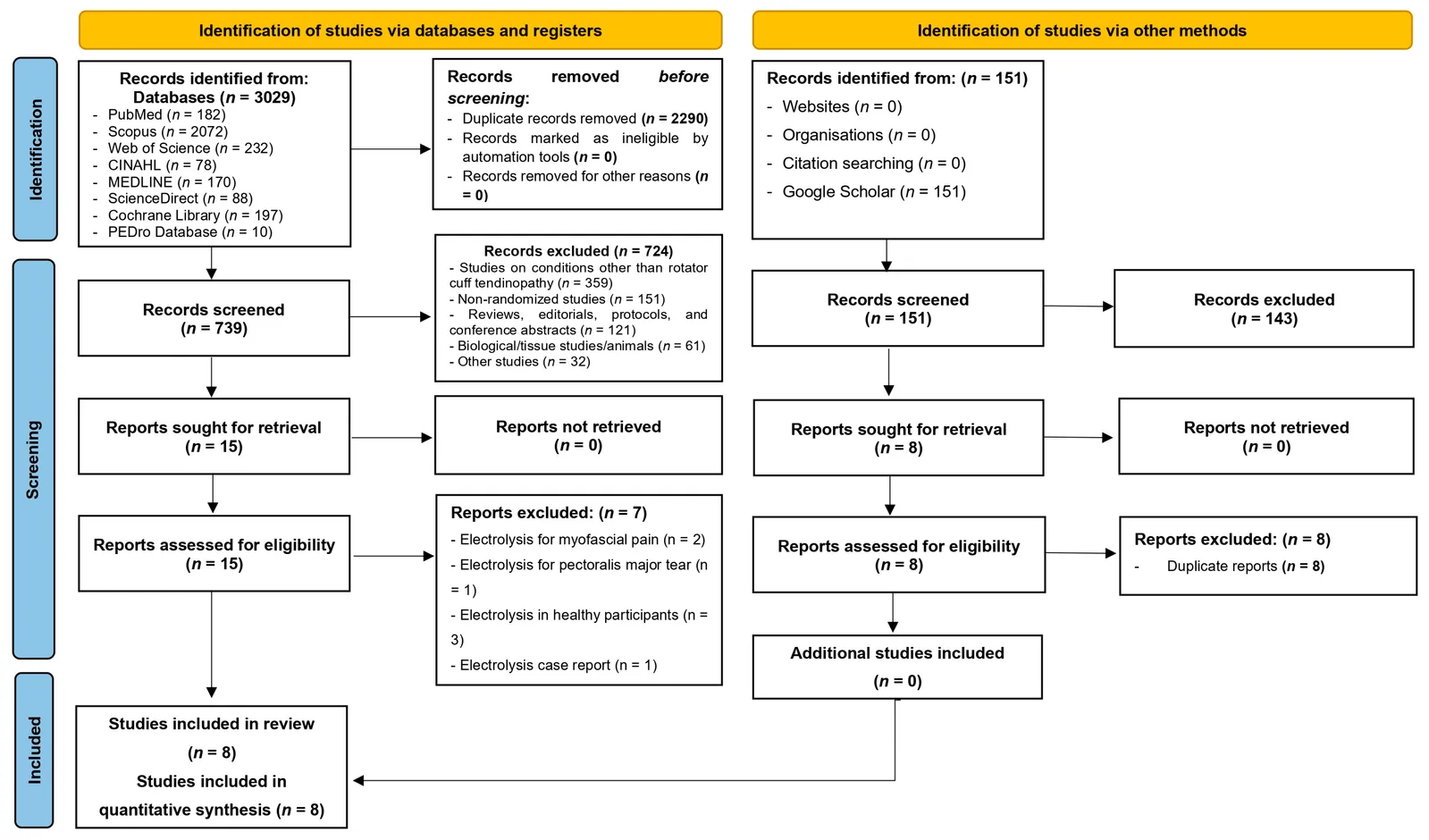
PRISMA 2020 flow diagram of the study selection process.

Seven full-text reports were excluded because they investigated conditions other than RC tendinopathy, including myofascial pain (*n* = 2) and pectoralis major tear (*n* = 1); enrolled healthy participants (*n* = 3); or were case reports (*n* = 1). Of the 151 records identified through Google Scholar, 143 were excluded during title and abstract screening. The remaining eight reports were assessed for eligibility and excluded because they duplicated reports already identified through the database searches. Therefore, Google Scholar did not contribute any additional studies to the review. The study selection process is presented in Figure 1, whereas Supplementary Table S1 details the complete search strategy.

### 3.2. RoB Assessment

**Figure 2:**
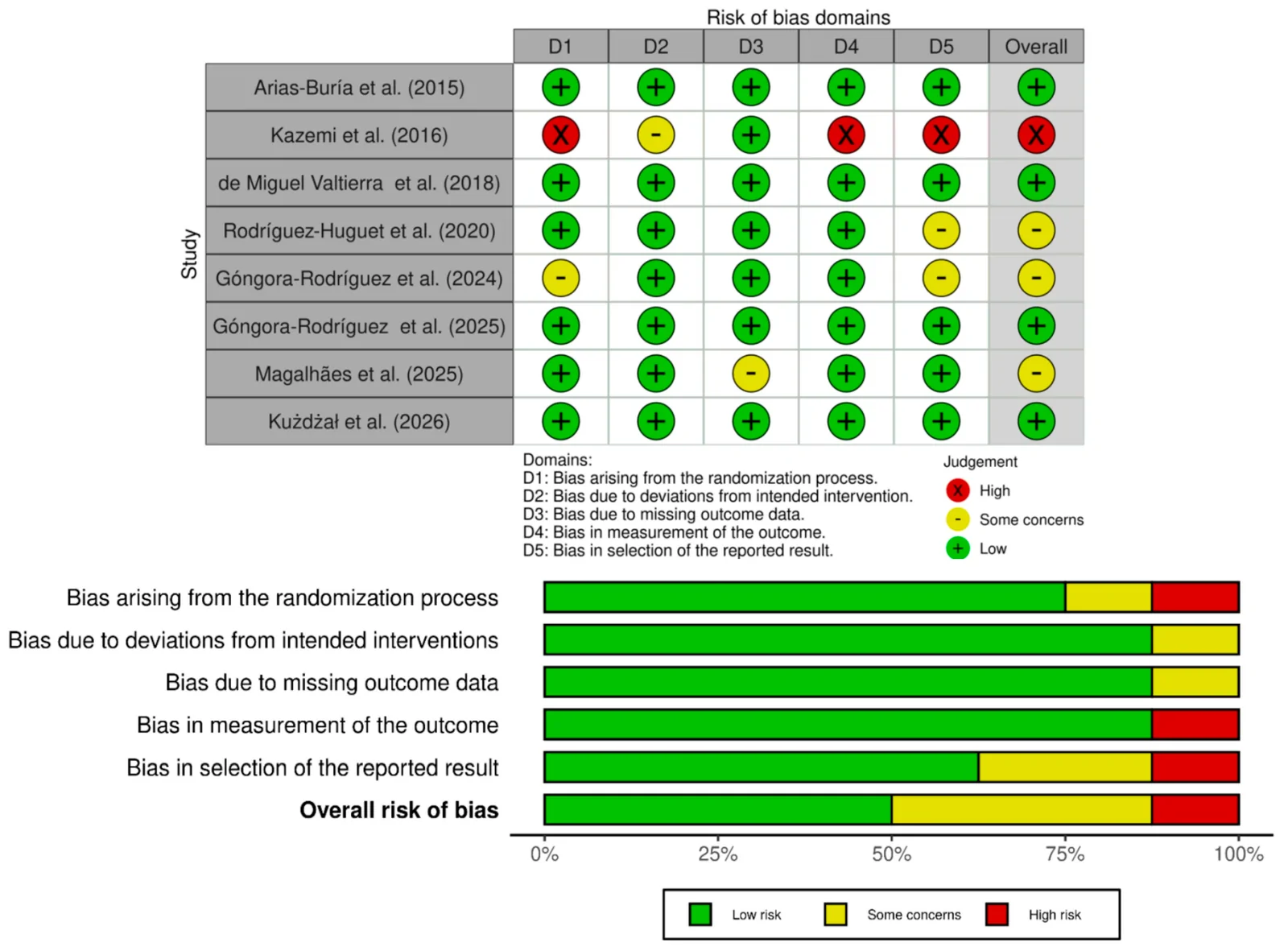
RoB Across Included RCTs.

Figure 2 summarizes the RoB 2 assessment of the included randomized controlled trials. Inter-reviewer agreement before consensus was substantial (Fleiss’ κ = 0.75, 95% CI 0.63–0.90) [28]. Overall, four of the eight studies (50.0%) were judged at low RoB, three (37.5%) were rated as having some concerns, and one (12.5%) was judged at high RoB [27]. At the domain level, low risk predominated for deviations from intended interventions (D2), missing outcome data (D3), and outcome measurement (D4). In contrast, selection of the reported result (D5) was the domain most commonly associated with methodological concerns.

### 3.3. Study Characteristics

Table 1 summarizes the characteristics of the eight included RCTs published between 2015 and 2026 [37,38,39,40,41,42,43,44]. Most studies were conducted in Spain (*n* = 6) [37,38,39,40,41,42], with one study each from Brazil [43] and Poland [44]. Overall, 330 participants were enrolled, including 173 men and 157 women, with a pooled mean age of 47.8 ± 11.9 years [37,38,39,40,41,42,43,44]. Diagnostic terminology varied across studies. Two RCTs enrolled participants diagnosed with subacromial pain/impingement syndrome [37,39], three specifically included supraspinatus tendinopathy [40,41,42], one included chronic rotator cuff tendinopathy [43], one included subscapularis tendinopathy [44], and one included symptomatic infraspinatus tendon involvement [38]. Across studies, eligibility was based on a symptomatic clinical presentation attributable to the rotator cuff, with ultrasonography or magnetic resonance imaging used as supportive diagnostic tools when reported. Detailed diagnostic and eligibility criteria for each included RCT are presented in Supplementary Table S3.

Six RCTs investigated MEL, predominantly using the EPTE^®^ (Therapeutic Percutaneous Electrolysis) technique (*n* = 5) [37,39,40,41,42], whereas one evaluated MEL using the MEP^®^ (Percutaneous Microelectrolysis) technique [43]. EL using the EPI^®^ (Intratissue Percutaneous Electrolysis) technique was investigated in two RCTs [38,44]. Across all experimental groups, 174 participants received EL or MEL (129 MEL and 45 EL), whereas 156 participants were allocated to comparator groups [37,38,39,40,41,42,43,44]. Only one RCT investigated EL as a stand-alone intervention [38]. In the remaining studies, EL or MEL was combined with conservative rehabilitation, most commonly therapeutic exercise, including eccentric exercise [37,40,41,42] or the Getting it Right: Addressing Shoulder Pain (GRASP) programme [43]. Additional co-interventions included manual therapy [39,44], percutaneous electrical nerve stimulation (PENS) [41,42], and extracorporeal shockwave therapy (ESWT) [44].

Most comparator interventions were active and included therapeutic exercise [37,39,43], manual therapy [39,44], dry needling [40], transcutaneous electrical nerve stimulation (TENS) and therapeutic ultrasound [41,42], and ESWT [44], delivered either alone or as part of multimodal rehabilitation.

Treatment sessions were delivered once weekly in all studies [37,38,39,40,41,42,43,44], with interventions ranging from 3 to 8 sessions (mean 5 sessions). Four-session treatment protocols were the most common (*n* = 4) [37,40,41,42].

PEDro scores for the included RCTs ranged from 5 to 9 (maximum score, 10) [37,38,39,40,41,42,43,44], with a mean score of 7.0 ± 1.4. Detailed PEDro assessments are provided in Supplementary Table S4 [26].

### 3.4. Assessment Time Points and Outcomes

Table 1 presents the assessment schedule and outcome measures used across the included RCTs. The timing of assessments differed among studies, although all trials collected baseline data (T0) and performed at least one post-treatment assessment between 3 and 8 weeks after the intervention began [37,38,39,40,41,42,43,44]. Two RCTs also incorporated interim assessments during treatment [37,38]. Follow-up assessments were reported in four RCTs [39,40,41,42]. The first follow-up assessment occurred within the first 3 months, whereas the longest available follow-up extended to 1 year [40]. Two studies assessed outcomes only at baseline and post-treatment [43,44].

Pain intensity was assessed in all eight RCTs using either the NPRS [37,39,40,41,42,43] or the VAS [38,44]. Disability was evaluated in six studies using the DASH questionnaire [37,39,41,42,44] and/or the SPADI [39,41,42,43]. PPT was assessed in four RCTs using algometry [39,40,42,44], whereas shoulder ROM was evaluated in four studies using goniometry [38,40,42,44]. Shoulder strength was measured in two RCTs using dynamometry [41,44].

Additional outcomes included tendon morphology assessed by ultrasonography [41,44], muscle activity measured by electromyography [41], tissue perfusion assessed by laser Doppler flowmetry [44], tendon stiffness measured using MyotonPRO^®^ (Myoton AS, Tallinn, Estonia) [44], tenascin-C concentrations measured using the Enzyme-Linked Immunosorbent Assay (ELISA) [44], self-reported improvement assessed using the GROC scale [39], global perceived effect [43], exercise adherence [43], and subscapularis clinical tests (Lift-off, Belly-press, and Bear-hug tests) [44].

### 3.5. Summary of Individual Study Findings

Table 1 summarizes the main findings and between-group effect estimates of the included RCTs. Pain intensity was evaluated in all eight RCTs using the NPRS [37,39,40,41,42,43] or VAS [38,44]. Between-group effects generally favored EL/MEL-based interventions, although the magnitude of the effect varied across studies and comparator interventions, with smaller and less precise estimates reported by Magalhães et al. [43] and Kużdżał et al. [44]. Shoulder-related disability was evaluated in six RCTs using the DASH [37,39,41,42,44] and/or SPADI [39,41,42,43], with between-group effects ranging from little or no additional benefit to substantial reductions in disability favoring electrolysis-based interventions.

Shoulder ROM was assessed in four RCTs [38,40,42,44], with between-group effects varying according to movement direction and comparator intervention. PPT was also evaluated in four RCTs [39,40,42,44]; three reported greater between-group improvements following electrolysis-based interventions [40,42,44], whereas de Miguel Valtierra et al. [39] reported estimates close to the null across measurement sites. Additional outcomes included shoulder strength, muscle activity, tendon morphology, tendon stiffness, tissue perfusion, and Tenascin-C concentrations [41,44]. Detailed study-level between-group estimates and 95% CIs are presented in Table 1.

At the individual-study level, a 36-participant RCT reported between-group differences favoring MEL plus eccentric exercise for pain (MD = 1.90 points; 95% CI: 0.70 to 3.10) and DASH disability (MD = 9.50 points; 95% CI: 1.90 to 17.20) [37]. In a 50-participant RCT, MEL was associated with between-group differences of 1.40 points for pain (95% CI: 0.40 to 2.40) and 12.50 points for SPADI (95% CI: 6.10 to 18.90), whereas estimates for DASH and PPT were small and imprecise [39]. A 36-participant trial reported a 1.99-point between-group difference in pain (95% CI: 0.55 to 3.44), together with higher PPT and greater external rotation following MEL compared with dry needling [40]. Larger effects were reported in two RCTs. In one trial (*n* = 50), between-group differences favored MEL-based treatment for pain (MD = 2.96 points; 95% CI: 1.86 to 4.06), DASH (MD = 33.16 points; 95% CI: 24.25 to 42.07), and SPADI (MD = 30.96 points; 95% CI: 22.23 to 39.69), together with improvements in shoulder strength and tendon morphology [41]. In another trial (*n* = 46), corresponding between-group differences were 3.61 points for pain (95% CI: 2.69 to 4.53), 36.83 points for DASH (95% CI: 28.18 to 45.48), and 35.35 points for SPADI (95% CI: 27.27 to 43.43), with additional effects on PPT and ROM [42].

By contrast, a 42-participant RCT found no clear additional benefit of MEL plus therapeutic exercise over therapeutic exercise alone for pain (MD = 1.23 points; 95% CI: −0.07 to 2.54) or SPADI (MD = 7.17 points; 95% CI: −7.02 to 21.35) [43]. In a 30-participant trial, little between-group difference was observed for pain (MD = 0.07; 95% CI: −0.64 to 0.77), although EL added to multimodal rehabilitation favored the intervention group for DASH disability (MD = 3.91 points; 95% CI: 1.97 to 5.84), PPT (MD = 10.01 kPa; 95% CI: 9.07 to 10.94), internal-rotation ROM (MD = 5.29°; 95% CI: 4.18 to 6.39), and internal-rotation strength (MD = 1.53 kgF; 95% CI: 1.15 to 1.92) [44]. Finally, a 40-participant trial comparing EL with no treatment reported its largest effects when both the infraspinatus tendon and myofascial trigger points were treated, including a 70-point between-group difference in VAS pain (95% CI: 64.39 to 75.61) and marked reductions in ROM restriction [38].

### 3.6. Electrolysis Intervention Characteristics

The technical characteristics of the electrolysis interventions are summarized in Table 2. Six of the eight included RCTs evaluated MEL [37,39,40,41,42,43], whereas two evaluated EL [38,44]. Five MEL studies used ultrasound guidance [37,39,40,41,42], whereas one was performed without imaging guidance [43]. Current intensity ranged from 350 to 900 µA (0.35–0.9 mA), application time from 40 to 90 s, and delivered electrical charge from 25.2 to 36 mC per session (mean, 28.1 ± 4.6 mC). Needle size ranged from 0.25 × 30 mm to 0.30 × 40 mm, and treatment protocols consisted of 4–6 sessions delivered once weekly.

Both EL studies were performed under ultrasound guidance [38,44]. Current intensity ranged from 1.5 to 6 mA, application time ranged from 4 s per application (three applications) to 180 s per application (three applications), needle size was selected according to participant anthropometry in one study and was 0.30 × 30 mm in the other, delivered electrical charge ranged from 72 to 810 mC per session, and treatment protocols consisted of 3–8 sessions delivered once weekly.

Overall, the electrolysis protocols varied across studies in terms of modality, current intensity, electrical charge, application time, needle characteristics, imaging guidance, and number of treatment sessions. Given the small number of available RCTs, it was not possible to perform meaningful subgroup analyses according to individual treatment parameters. These differences should therefore be considered when interpreting the pooled effect estimates and the heterogeneity observed across studies.

### 3.7. Meta-Analysis by Outcome

Given the clinical heterogeneity across studies in electrolysis protocols, co-interventions, comparators, and follow-up periods, studies were grouped according to outcome, intervention–comparator structure, and assessment time point. When sufficient data were available, subgroup analyses were conducted to account for clinically relevant differences between comparisons. An overall pooled effect was also estimated when studies were considered sufficiently comparable and was interpreted together with the subgroup effects and measures of between-study heterogeneity.

**Figure 3:**
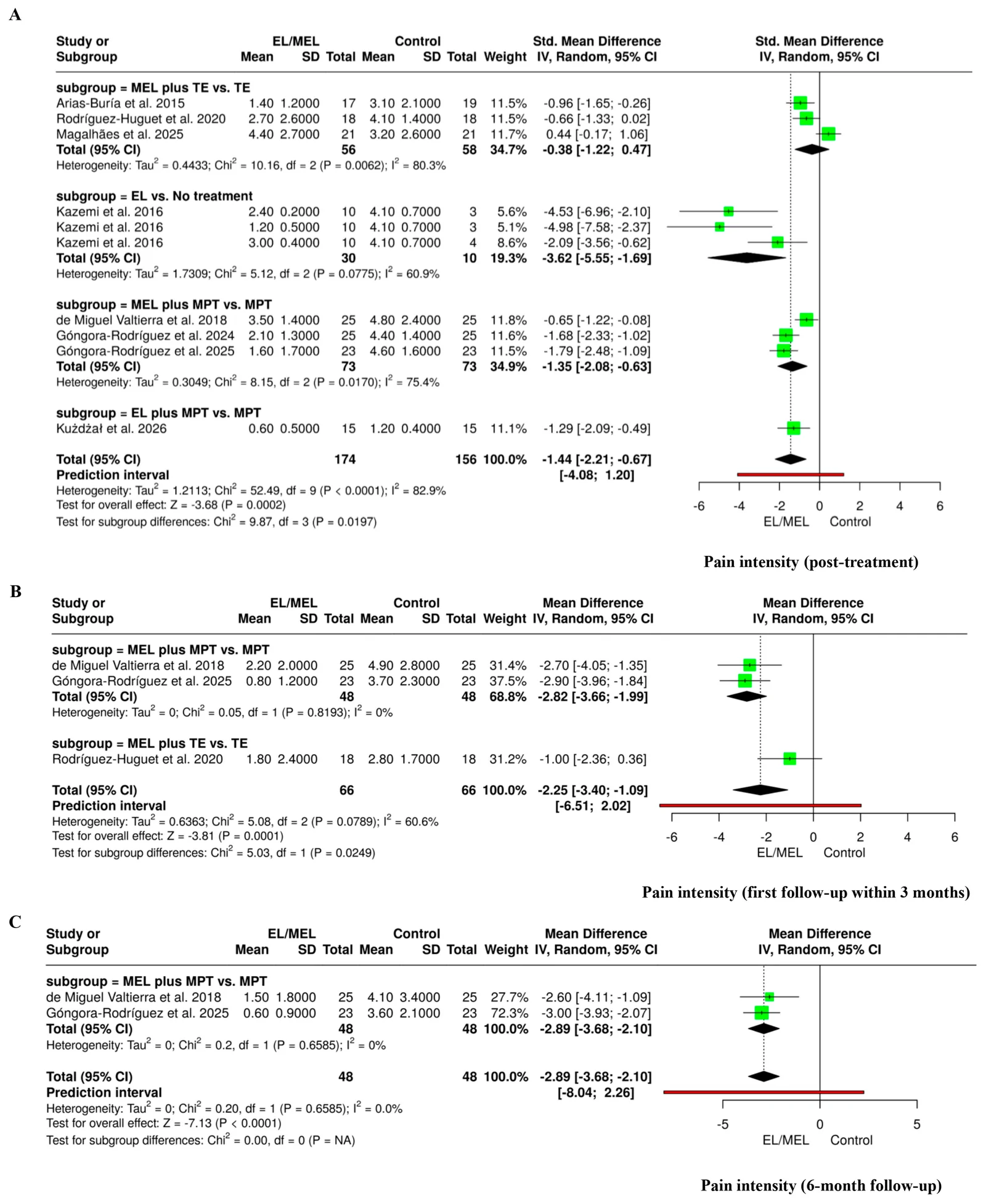
Forest plots generated from the present meta-analysis using data from the included RCTs, illustrating the effects of EL and MEL on pain intensity compared with control interventions. (**A**) Post-treatment pain intensity assessed using the NPRS and VAS according to comparator intervention [37,38,39,40,41,42,43,44]. (**B**) Pain intensity assessed using the NPRS at the first follow-up within the first 3 months after treatment [39,40,42]. (**C**) Pain intensity assessed using the NPRS at the 6-month follow-up [39,42]. Effect estimates are reported as SMDs for post-treatment analyses and MDs for follow-up analyses, with 95% confidence intervals derived from random-effects models. Negative effect estimates indicate lower pain intensity scores in the EL/MEL groups relative to the control groups.

*Pain intensity (post-treatment).* The pooled analysis of pain intensity is shown in Figure 3A and includes EL and MEL compared with control interventions [37,38,39,40,41,42,43,44]. The meta-analysis incorporated 10 comparisons from eight RCTs, with 174 participants allocated to EL/MEL and 156 to control groups. Pain intensity was assessed using the NPRS [37,39,40,41,42,43] and the VAS [38,44]. Because different pain intensity scales (NPRS and VAS) were used across studies, pooled effects were calculated as SMDs using a random-effects inverse-variance model. The multi-arm trial by Kazemi et al. [38], which included three EL intervention groups sharing a single control group, was handled according to Cochrane recommendations by proportionally dividing the shared control group across the three pairwise comparisons to avoid double-counting participants [20]. Subgroup analyses according to comparator intervention showed significant reductions in pain intensity favoring EL over no treatment (SMD = −3.62; 95% CI: −5.55 to −1.69; *p* < 0.01) [38] and MEL combined with multimodal physiotherapy (PENS, therapeutic exercise, and manual therapy) compared with multimodal physiotherapy alone (SMD = −1.35; 95% CI: −2.08 to −0.63; *p* < 0.01) [39,41,42]. No significant differences were observed between MEL combined with therapeutic exercise and therapeutic exercise alone (SMD = −0.38; 95% CI: −1.22 to 0.47; *p* = 0.33) [37,40,43]. EL combined with multimodal physiotherapy (ESWT, manual therapy and therapeutic exercise) also resulted in greater reductions in pain intensity than multimodal physiotherapy alone (SMD = −1.29; 95% CI: −2.09 to −0.49; *p* < 0.01), although this subgroup included a single RCT [44].

The overall pooled analysis showed a significant reduction in pain intensity favoring the EL/MEL groups compared with the control groups (SMD = −1.44; 95% CI: −2.21 to −0.67; *p* < 0.05). According to the predefined Hedges’ g thresholds [33], the pooled effect size was large. Between-study heterogeneity was substantial (χ^2^ = 52.49, *p* < 0.01; τ^2^ = 1.21; I^2^ = 82.9%) [20], with a prediction interval of −4.08 to 1.20. The test for subgroup differences was statistically significant (χ^2^ = 9.87, df = 3, *p* = 0.0197).

The certainty of the evidence for post-treatment pain intensity was low (F). Pain intensity was predefined as a critical outcome for clinical decision-making [30,45].

*Pain intensity (follow-up).* Figure 3B,C present the pooled effects of EL and MEL on pain intensity at the first follow-up assessment, conducted within the first 3 months after treatment, and at the 6-month follow-up, respectively. At the first follow-up assessment, three RCTs were included in the quantitative synthesis [39,40,42], comprising 66 participants in the EL/MEL groups and 66 in the control groups. Pain intensity was assessed using the NPRS in all included studies [39,40,42]. Because all studies used the NPRS, pooled effects were calculated as MDs using inverse-variance models. The overall pooled analysis favored the EL/MEL groups, with a reduction in pain intensity of 2.25 points compared with the control groups (MD = −2.25; 95% CI: −3.40 to −1.09; *p* < 0.01). Between-study heterogeneity was moderate to substantial (χ^2^ = 5.08, *p* = 0.08; τ^2^ = 0.64; I^2^ = 60.6%) [20], with a prediction interval of −6.51 to 2.02.

At the 6-month follow-up, two RCTs including 48 participants in the MEL groups and 48 in the control groups contributed to the quantitative synthesis [39,42]. The overall pooled analysis showed a significant reduction in pain intensity favoring the MEL groups compared with the control groups (MD = −2.89; 95% CI: −3.68 to −2.10; *p* < 0.01). Between-study heterogeneity was not detected (χ^2^ = 0.20, *p* = 0.66; τ^2^ = 0.00; I^2^ = 0.0%) [20], with a prediction interval of −8.04 to 2.26.

The certainty of the evidence was low for the overall first follow-up within the first 3 months and moderate at the 6-month follow-up (Table 3) [30,45].

**Figure 4:**
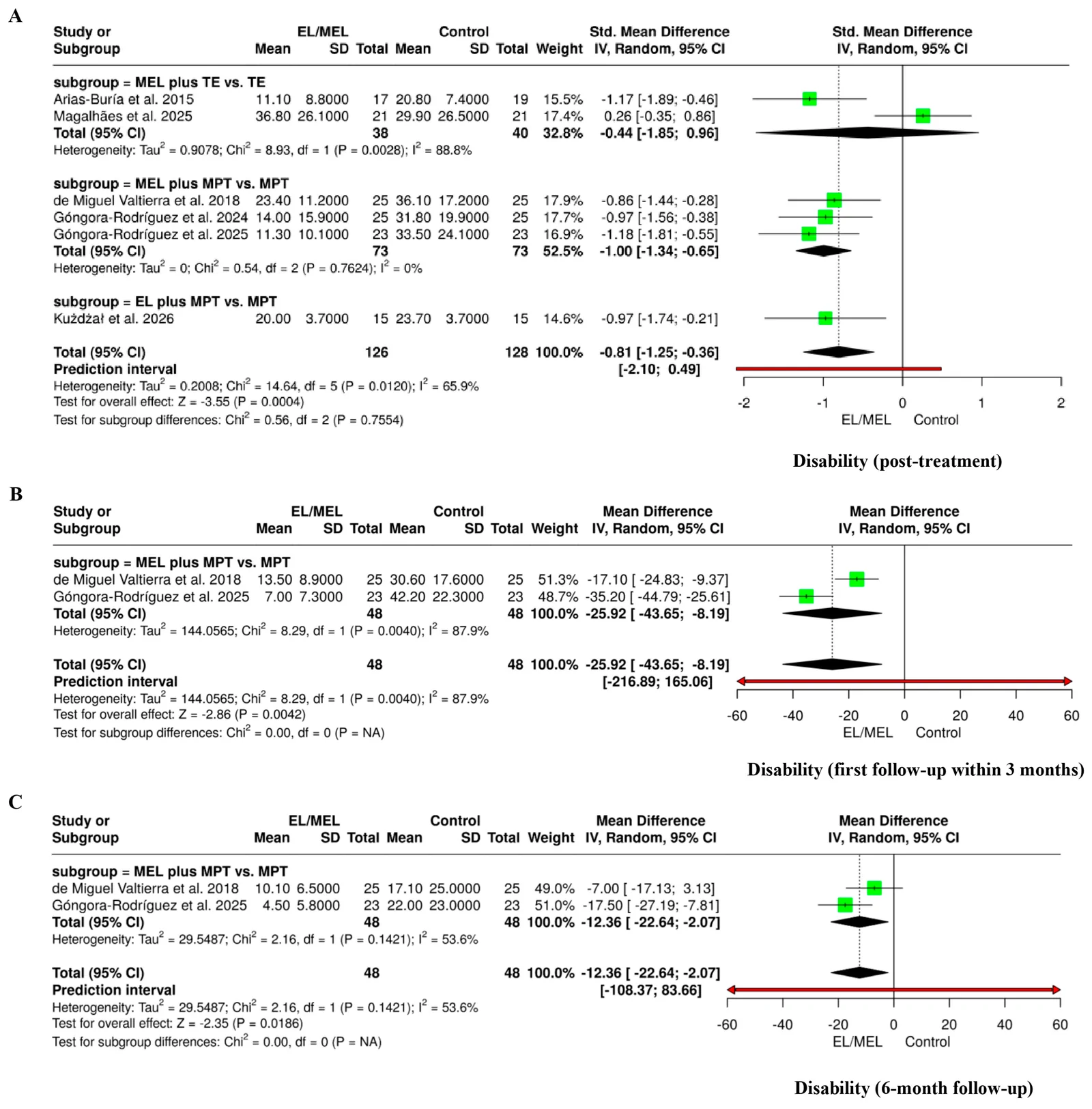
Forest plots generated from the present meta-analysis using data from the included RCTs, illustrating the effects of EL and MEL on disability compared with control interventions. (**A**) Post-treatment disability assessed using the DASH questionnaire and the SPADI according to comparator intervention [37,39,41,42,43,44]. (**B**) Disability assessed using the SPADI at the 3-month follow-up [39,42]. (**C**) Disability assessed using the SPADI at the 6-month follow-up [39,42]. Effect estimates are reported as SMDs for post-treatment analyses and MDs for follow-up analyses, with 95% confidence intervals derived from random-effects models. Negative effect estimates indicate lower disability scores in the EL/MEL groups relative to the control groups.

*Disability (post-treatment).* Figure 4A presents the meta-analysis of disability comparing EL and MEL with control interventions [37,39,41,42,43,44]. Six comparisons from six RCTs were included in the quantitative synthesis, including 126 participants in the EL/MEL groups and 128 in the control groups. Disability was assessed using the DASH questionnaire [37,39,41,42,44] and the SPADI [39,42]. Because different disability scales (DASH and SPADI) were used across studies, pooled effects were calculated as SMDs using a random-effects inverse-variance model. Subgroup analyses according to comparator intervention showed a significant reduction in disability favoring MEL combined with manual therapy compared with manual therapy alone (SMD = −1.00; 95% CI: −1.34 to −0.65; *p* < 0.01). No significant differences were observed between MEL combined with therapeutic exercise and therapeutic exercise alone (SMD = −0.44; 95% CI: −1.85 to 0.96; *p* = 0.43). EL combined with multimodal physiotherapy also favored EL over multimodal physiotherapy alone (SMD = −0.97; 95% CI: −1.74 to −0.21; *p* < 0.05), although this subgroup included a single RCT [44].

The pooled estimate showed a significant reduction in disability favoring the EL/MEL groups compared with the control groups (SMD = −0.81; 95% CI: −1.25 to −0.36; *p* < 0.01). According to the predefined Hedges’ g thresholds [33], the pooled effect size was large. Between-study heterogeneity was substantial (χ^2^ = 14.64, *p* < 0.05; τ^2^ = 0.20; I^2^ = 65.9%) [20], with a prediction interval of −2.10 to 0.49. Subgroup differences were not statistically significant (χ^2^ = 0.56, df = 2, *p* = 0.7554).

The certainty of the evidence for overall post-treatment disability was low (Table 3). Shoulder-related disability was predefined as a critical outcome for clinical decision-making [30,45].

Disability (follow-up). Figure 4B,C present the pooled effects of MEL on disability at the 3- and 6-month follow-up assessments [39,42]. At the 3-month follow-up, two RCTs comprising 48 participants in the MEL groups and 48 in the control groups were included in the quantitative synthesis. Disability was assessed using the SPADI in both studies. Because both studies used the same disability scales, pooled effects were calculated as MDs using a random-effects inverse-variance model. The overall pooled analysis showed a significant reduction in disability favoring the MEL groups compared with the control groups (MD = −25.92; 95% CI: −43.65 to −8.19; *p* < 0.01). Between-study heterogeneity was substantial (χ^2^ = 8.29, *p* < 0.01; τ^2^ = 144.06; I^2^ = 87.9%) [20], with a prediction interval of −216.9 to 165.1.

At the 6-month follow-up, the same two RCTs comprising 48 participants in the MEL groups and 48 in the control groups contributed to the quantitative synthesis. The overall pooled analysis showed a significant reduction in disability favoring the MEL groups compared with the control groups (MD = −12.36; 95% CI: −22.64 to −2.07; *p* < 0.05). Between-study heterogeneity was moderate (χ^2^ = 2.16, *p* = 0.14; τ^2^ = 29.55; I^2^ = 53.6%) [20], with a prediction interval of −108.4 to 83.7.

The certainty of the evidence for disability at both the 3- and 6-month follow-up assessments was low (Table 3) [30,45].

**Figure 5:**
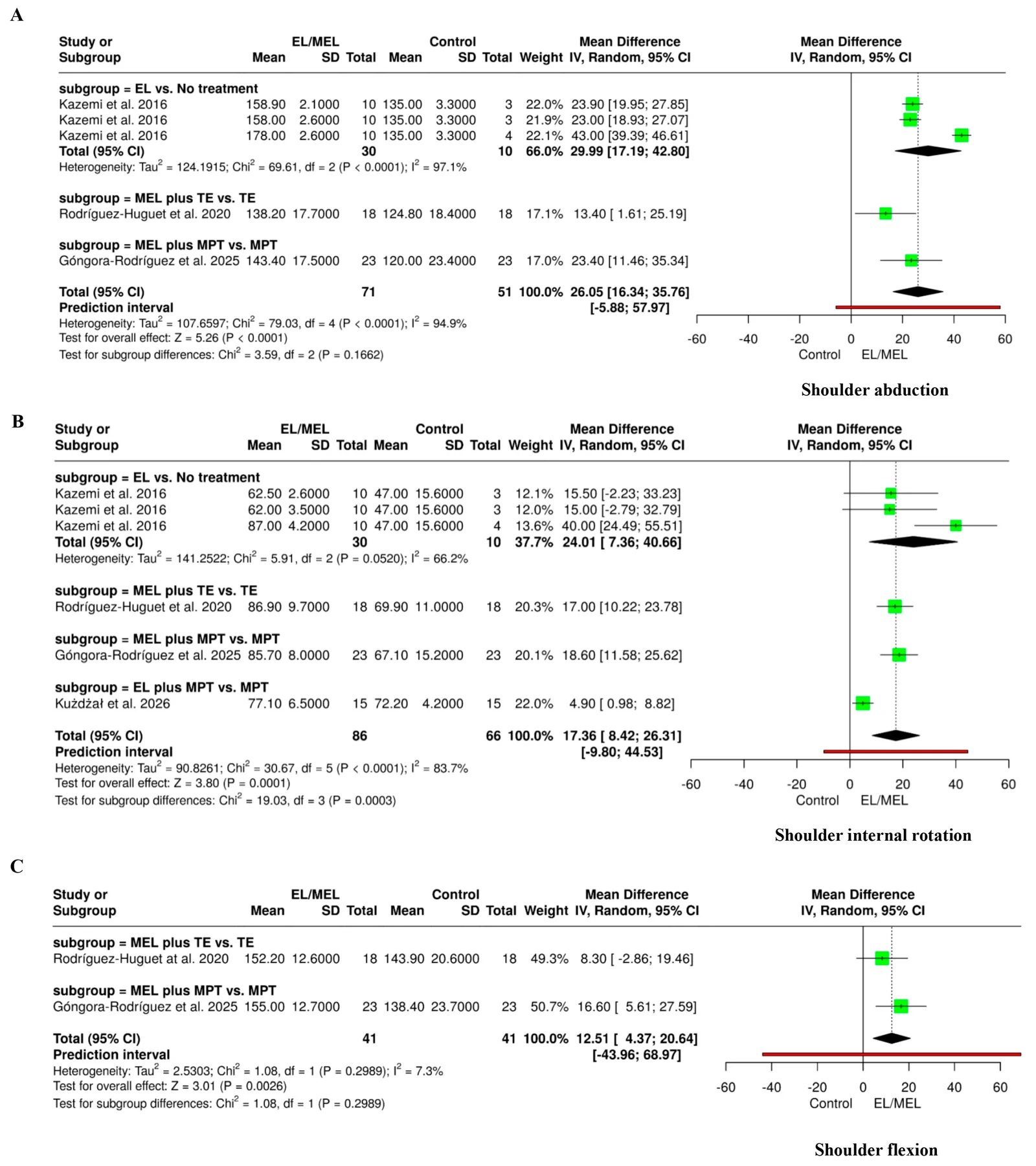
Forest plots generated from the present meta-analysis using data from the included RCTs, illustrating the effects of EL and MEL on shoulder ROM compared with control interventions. (**A**) Shoulder abduction [38,40,42]. (**B**) Shoulder internal rotation [38,40,42,44]. (**C**) Shoulder flexion [40,42]. Effect estimates are reported as MDs with 95% confidence intervals derived from random-effects models. Positive MD values indicate greater ROM in the EL/MEL groups relative to the control groups.

*Shoulder ROM (post-treatment).* Figure 5A–C present the meta-analyses of shoulder ROM comparing EL and MEL with control interventions. Shoulder ROM was measured using goniometry in all included RCTs. Because all studies reported shoulder ROM in degrees, pooled effects were calculated as MDs using random-effects inverse-variance models. External rotation was not quantitatively pooled because of marked clinical and statistical heterogeneity and inconsistent effects across comparisons and was therefore summarized narratively.

Shoulder abduction (Figure 5A) was evaluated in five comparisons from three RCTs, comprising 71 participants in the EL/MEL groups and 51 in the control groups [38,40,42]. The overall pooled analysis showed significantly greater improvements in shoulder abduction favoring the EL/MEL groups compared with the control groups (MD = 26.05°; 95% CI: 16.34 to 35.76; *p* < 0.01). Heterogeneity was considerable (χ^2^ = 79.03, *p* < 0.01; τ^2^ = 107.66; I^2^ = 94.9%), with a prediction interval of −5.88° to 57.97°. Subgroup differences were not statistically significant (χ^2^ = 3.59, df = 2, *p* = 0.1662). Given the high heterogeneity and prediction interval crossing the null, the pooled estimate should be interpreted cautiously.

Shoulder internal rotation (Figure 5B) was evaluated in six comparisons from four RCTs, comprising 86 participants in the EL/MEL groups and 66 in the control groups [38,40,42,44]. The overall pooled analysis demonstrated significantly greater improvements in internal rotation favoring the EL/MEL groups (MD = 17.36°; 95% CI: 8.42 to 26.31; *p* < 0.01). Heterogeneity was considerable (χ^2^ = 30.67, *p* < 0.01; τ^2^ = 90.83; I^2^ = 83.7%), with a prediction interval of −9.80° to 44.53°. The test for subgroup differences was statistically significant (χ^2^ = 19.03, df = 3, *p* = 0.0003), indicating that the magnitude of the effect differed according to the comparator intervention. Accordingly, the overall pooled estimate should be interpreted with caution.

Shoulder flexion (Figure 5C) was evaluated in two comparisons from two RCTs, comprising 41 participants in the EL/MEL groups and 41 in the control groups [40,42]. The pooled analysis demonstrated significantly greater improvements in shoulder flexion favoring the EL/MEL groups (MD = 12.51°; 95% CI: 4.37 to 20.64; *p* < 0.01). Heterogeneity was low (χ^2^ = 1.08, *p* = 0.30; τ^2^ = 2.53; I^2^ = 7.3%), although the prediction interval was wide (−43.96° to 68.97°), reflecting the limited number of contributing RCTs. Subgroup differences were not statistically significant (χ^2^ = 1.08, df = 1, *p* = 0.2898).

Shoulder external rotation was evaluated in three RCTs [38,40,42]. Given the limited number of studies and the very high heterogeneity observed in the initial quantitative synthesis (I^2^ = 98.0%), together with differences in outcome reporting, intervention protocols, and comparators, findings were summarized narratively. Kazemi et al. [38] assessed external rotation restriction and reported post-treatment values of 29.0 ± 3.94°, 29.5 ± 4.38°, and 0 ± 0° for the trigger-point, tendon, and combined trigger-point-plus-tendon EL groups, respectively, versus 50.0 ± 4.08° in the control group; the 0 ± 0° value represented absence of residual restriction rather than missing data. All intervention groups improved significantly, with the greatest improvement in the combined-treatment group (*p* < 0.001). Rodríguez-Huguet et al. [40] reported greater post-treatment external rotation following MEL than control (86.83 ± 5.88° vs. 70.33 ± 12.27°), with a significant time-by-group interaction. Góngora-Rodríguez et al. [42] similarly found greater post-treatment external rotation with MEL-based treatment (84.04 ± 10.77° vs. 70.96 ± 17.14°; between-group difference in change = 12.70°; *p* = 0.006).

The certainty of the evidence for post-treatment shoulder ROM ranged from low to moderate across comparisons (Table 3). However, these ratings were based on a limited number of RCTs and should therefore be interpreted cautiously. Shoulder ROM was predefined as an important outcome for clinical decision-making [30,45].

*PPT (post-treatment).* Pressure pain threshold was assessed using pressure algometry in four RCTs [39,40,42,44]. Given the limited number of RCTs, substantial clinical heterogeneity in electrolysis protocols, co-interventions, and comparator treatments, and considerable inconsistency across study estimates, quantitative pooling was considered inappropriate, and findings were summarized narratively. Three RCTs reported significantly greater improvements in PPT following electrolysis-based interventions compared with their respective control interventions [40,42,44]. Rodríguez-Huguet et al. [40] reported greater improvements in proximal, middle, and distal PPT following MEL combined with eccentric exercise compared with dry needling and eccentric exercise. Góngora-Rodríguez et al. [42] similarly reported significantly greater post-treatment PPT improvements following MEL combined with PENS and eccentric exercise compared with conventional electrotherapy and eccentric exercise. Kużdżał et al. [44] found a significant between-group difference in PPT favoring EL added to multimodal rehabilitation (between-group difference in change, 10.01 kPa; 95% CI: 9.07 to 10.94; *p* < 0.001). In contrast, de Miguel Valtierra et al. [39] found no significant between-group differences in PPT after adding MEL to manual therapy and exercise.

### 3.8. Sensitivity Analysis

**Figure 6:**
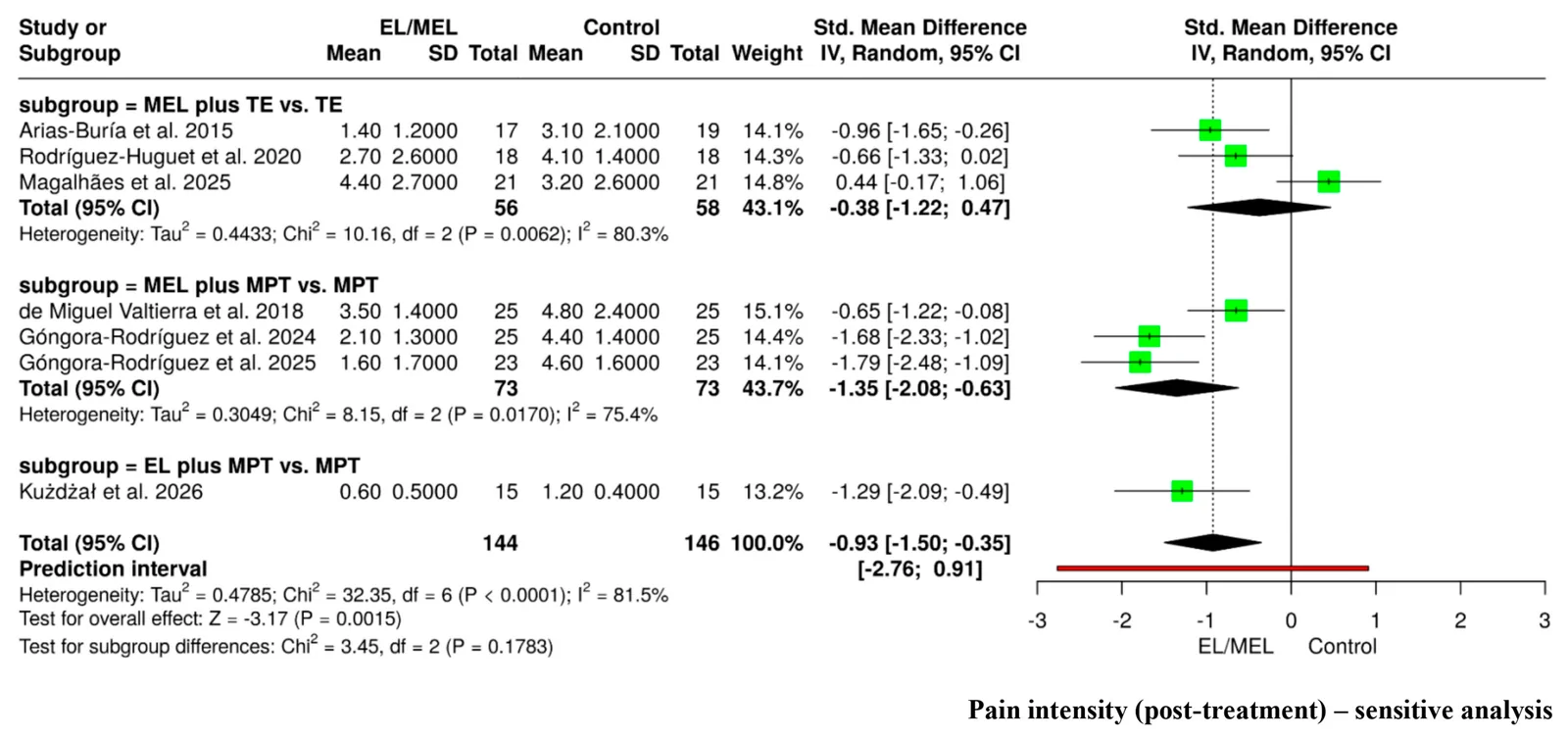
Sensitivity analysis of the effect of EL and MEL on post-treatment pain intensity after excluding the RCT judged to have a high overall RoB (Kazemi et al. [38]). Effect sizes are presented as SMDs with 95% confidence intervals estimated using a random-effects model. Negative SMD values favor the EL/MEL groups over the control interventions.

Sensitivity analysis was performed for post-treatment pain intensity by excluding the RCT judged to have a high overall RoB (Kazemi et al. [38]). After exclusion of this study, the pooled effect remained statistically significant (SMD = −0.93; 95% CI: −1.50 to −0.35; χ^2^ = 32.35, *p* < 0.0001; τ^2^ = 0.48; I^2^ = 81.5%), with a prediction interval ranging from −2.76 to 0.91 (Figure 6). Although the magnitude of the pooled effect was attenuated, the direction and statistical significance of the effect were maintained. A sensitivity analysis was not performed for shoulder-related disability because none of the RCTs contributing data to this outcome was judged to have a high overall RoB.

### 3.9. Certainty of Evidence

The certainty of evidence for the meta-analyzed outcomes is summarized in Table 3. Overall, certainty ranged from low to moderate, and no outcome was rated as high-certainty evidence. The overall post-treatment effects for pain intensity and shoulder-related disability were rated as low certainty, primarily because of inconsistency and imprecision. For pain intensity, certainty remained low at the first follow-up within the first 3 months and was moderate at the 6-month follow-up. For shoulder ROM, certainty was low for abduction and internal rotation and moderate for flexion.

### 3.10. Adverse Events

Adverse-event findings were summarized descriptively (Table 1). Arias-Buría et al. [37] reported local post-treatment soreness in 6 of 17 participants receiving MEL, whereas de Miguel Valtierra et al. [39] reported muscle soreness in 6 of 25 participants. Magalhães et al. [43] reported non-serious adverse effects in 12 of 21 participants receiving MEL plus exercise and in 14 of 21 participants receiving exercise alone, with no unexpected serious adverse effects. The remaining trials did not provide sufficiently detailed adverse-event reporting or ascertainment procedures to distinguish confidently between zero events and absence of reporting. Therefore, absence of reported adverse events was not interpreted as evidence of zero events. Given the available RCT evidence, adverse-event incidence was not quantitatively estimated.

### 3.11. Clinical Relevance

Consistent with Cochrane guidance, clinical relevance was evaluated primarily from the overall pooled estimates derived from the meta-analyses, which provide a quantitative summary of intervention effects across studies [20,45,46,47]. Based on the thresholds proposed by Swinton et al. [33], the post-treatment effects for pain intensity (SMD = −1.44; 95% CI: −2.21 to −0.67) and disability (SMD = −0.81; 95% CI: −1.25 to −0.36) were classified as large. For outcomes pooled as MDs, the estimates were compared with published MCID values. Pain reductions of 2.25 NPRS points at the first follow-up within 3 months and 2.89 points at 6 months were within or above the reported MCID range of 1.5–2.5 points [48]. For disability, the pooled SPADI reduction of 25.92 points at 3 months exceeded the reported MCID range of 18–25 points, whereas the 12.36-point reduction at 6 months remained below this range [22,48]. These findings should also be interpreted in the context of the between-study heterogeneity observed in several pooled analyses, particularly for post-treatment pain and disability and short-term disability.

### 3.12. Publication Bias

Publication bias was not assessed because all meta-analyses included fewer than 10 studies, precluding reliable evaluation using funnel plots or statistical tests for small-study effects, in accordance with current methodological recommendations [20,36,49].

## 4. Discussion

This systematic review and meta-analysis provides a comprehensive condition-specific synthesis of RCTs evaluating the effectiveness of EL and MEL for RC tendinopathy and summarizing reported adverse events. Overall, the available evidence suggests that electrolysis-based interventions may improve pain intensity and shoulder-related disability, with potential benefits for selected secondary outcomes, particularly ROM [37,38,39,40,41,42,43,44]. However, substantial clinical and methodological heterogeneity across the included RCTs, including differences in treatment protocols, co-interventions, comparator interventions, participant characteristics, and outcome assessment, warrants cautious interpretation of these findings.

### 4.1. Electrolysis and Pain Intensity

Overall, electrolysis was associated with significant reductions in pain intensity compared with control interventions, supporting its potential role as an adjunctive treatment for individuals with RC tendinopathy. These findings were observed despite substantial clinical heterogeneity across studies, including differences in electrolysis protocols, comparator interventions, and rehabilitation approaches. They are consistent with previous systematic reviews reporting beneficial effects of electrolysis on pain in musculoskeletal disorders and tendinopathies [13,14,15,50]. The present review extends the available evidence by incorporating recently published RCTs, including studies evaluating MEL [37,39,40,41,42,43].

The observed analgesic effects are biologically plausible and may be explained by the combined interaction of mechanical, electrochemical, and neurophysiological mechanisms. Unlike conventional dry needling, electrolysis combines the mechanical stimulus of needle insertion with the application of direct current (galvanic), with the acupuncture needle serving as the cathode [12,16,51]. At this site, electrolysis of water generates localized alkaline products, including sodium and potassium hydroxides (NaOH and KOH), together with molecular hydrogen. These localized electrochemical reactions may contribute to a controlled inflammatory response, extracellular matrix remodeling, and restoration of tendon homeostasis [10,12,16,52]. In addition, electrolysis may share several neurophysiological mechanisms described for other needle-based interventions, including modulation of peripheral nociceptive input, reduced pain-related muscle inhibition, increased pressure pain thresholds, and activation of endogenous pain inhibitory pathways [12,48,53,54]. Together, these complementary mechanisms may contribute to the pain reductions observed across the included studies.

An important finding of this review was that the magnitude of pain reduction appeared to vary according to the rehabilitation approach. The largest treatment effects were generally observed when EL/MEL was combined with therapeutic exercise and multimodal physiotherapy, whereas studies evaluating electrolysis modalities alone or against active conservative interventions tended to show smaller between-group differences [37,39,41,42,43]. These findings are consistent with current clinical practice guidelines, which identify therapeutic exercise as the cornerstone of conservative management for RC tendinopathy, while recognizing that adjunctive interventions may reduce pain, improve tolerance to tendon-loading exercises, and facilitate rehabilitation [1,39,50,55].

Interestingly, both EL and MEL were associated with favorable analgesic outcomes despite substantial differences in current intensity, treatment duration, and electrical charge delivered. Although MEL typically uses microampere currents and lower electrical charges than conventional EL, pain reductions were observed with both modalities [10]. These findings suggest that achieving a sufficient biological stimulus may be more important than maximizing the delivered electrical charge, as increasing the electrical dose beyond an effective biological threshold may not necessarily enhance therapeutic outcomes [55]. However, this hypothesis remains speculative because no RCT has directly compared EL and MEL using standardized protocols or evaluated dose–response relationships.

Clinically, the reduction in pain may represent a meaningful benefit; however, this finding should be interpreted with caution because it is based on a small number of contributing RCTs. Although the post-treatment meta-analysis was synthesized using SMDs, precluding direct comparison with published MCID thresholds, the follow-up analyses were pooled using MDs expressed on the NPRS. Pain improved by 2.3 points on the NPRS at the first follow-up within the first 3 months and by 2.9 points at the 6-month follow-up, values that fall within or above the reported MCID range of 1.5 to 2.5 points for individuals with RC-related shoulder pain and subacromial pain syndrome [48,56,57]. These improvements may also be meaningful from the patient’s perspective, consistent with the growing emphasis on the Patient Acceptable Symptom State (PASS) in upper-extremity rehabilitation. Although PASS thresholds for NRS pain ranging from 0.73 to 2.1 have been reported in postoperative shoulder, hand, and upper-extremity populations [57,58], their applicability to conservatively managed RC tendinopathy remains uncertain. Therefore, the observed pain reductions suggest a potentially clinically relevant benefit, but this interpretation should not be considered definitive given the limited number of RCTs and the heterogeneity of the available evidence. When integrated into rehabilitation programs centered on progressive therapeutic exercise, EL/MEL may represent a potential adjunctive intervention for pain management, consistent with current clinical practice guidelines that identify exercise as the cornerstone of conservative treatment for RC tendinopathy [1].

### 4.2. Electrolysis and Disability

Restoring function is a primary goal in the management of RC tendinopathy, as pain reduction does not necessarily translate into better performance of daily, occupational, or recreational activities. Consequently, disability is commonly assessed using patient-reported outcome measures (PROMs), which capture patients’ perceptions of symptoms and functional limitations. Among these, the SPADI and DASH are among the most frequently used and best-validated instruments for shoulder disorders, including RC tendinopathy [22,23,56,57,58,59].

Our findings showed that electrolysis-based interventions were associated with statistically significant improvements in self-reported disability compared with control interventions. However, the magnitude and robustness of these effects should be interpreted cautiously because of variability in rehabilitation protocols and outcome measures across the included RCTs [37,38,39,40,41,42,43,44], together with the limited number of studies contributing to some analyses. Overall, the pooled estimates favored electrolysis-based interventions, suggesting a potential benefit for shoulder-related function beyond pain reduction, although the clinical relevance of these improvements cannot be considered definitive.

The mechanisms underlying these functional improvements are likely multifactorial. By reducing pain, electrolysis-based interventions may facilitate participation in progressive therapeutic exercise and improve patients’ ability to perform functional activities, ultimately contributing to better disability outcomes. These findings are consistent with previous studies reporting greater improvements in shoulder pain and function when electrolysis was combined with exercise-based rehabilitation than with exercise alone, reinforcing the role of electrolysis-based interventions as adjuncts rather than standalone treatments [1,10,37,39].

From a clinical perspective, these findings should be interpreted cautiously. Because the primary disability meta-analysis combined SPADI and DASH outcomes using SMDs, direct comparison with published MCID values was not possible [59,60]. However, follow-up analyses pooled as MDs for SPADI showed improvements that approached or exceeded the reported MCID of 18–25 points for individuals with subacromial pain syndrome [22,48]. As these analyses included only a small number of RCTs, further high-quality studies using standardized disability measures and longer follow-up periods are warranted.

Overall, the available evidence suggests that electrolysis-based interventions may improve both pain and self-reported disability, supporting their use as adjuncts to rehabilitation programs centered on progressive therapeutic exercise [55].

### 4.3. Electrolysis and Other Clinical Outcomes

Among the secondary outcomes, improvements in ROM are particularly relevant because adequate shoulder mobility is essential for performing activities of daily living and overhead tasks. Restoration of glenohumeral ROM contributes not only to arm elevation but also to minimizing compensatory scapulothoracic movement patterns, making it an important component of functional recovery in individuals with RC tendinopathy [61,62].

Electrolysis-based interventions were associated with statistically significant improvements in shoulder abduction, internal rotation, and flexion. However, the clinical relevance and robustness of these effects should be interpreted cautiously because some analyses were based on a limited number of RCTs and showed substantial heterogeneity. Because these outcomes were pooled using MDs, the effects can be interpreted directly in degrees, facilitating comparison with recently proposed MCID values for shoulder ROM [63]. Although pain reduction may have contributed to the observed improvements, greater tolerance to movement and participation in progressive therapeutic exercise may also have facilitated recovery of shoulder mobility, supporting the potential role of electrolysis-based interventions as adjuncts rather than standalone treatments [1,39]. Improvements in shoulder mobility could, in turn, facilitate reaching, dressing, grooming, and overhead activities, which are commonly impaired in individuals with RC tendinopathy [1,5,61,62,63].

PPT findings were inconsistent across the four RCTs and were therefore summarized narratively. Three studies reported greater improvements in PPT following electrolysis-based interventions, whereas one study found no significant between-group difference. Although PPT is widely recognized as a clinically relevant measure of mechanical pain sensitivity in individuals with musculoskeletal shoulder disorders [64], the limited number of RCTs and the clinical and methodological heterogeneity across studies preclude definitive conclusions regarding the effects of electrolysis-based interventions on this outcome.

### 4.4. Adverse-Event Reporting

Treatment safety is an important consideration when selecting adjunctive interventions for individuals with RC tendinopathy because electrolysis is a percutaneous invasive procedure. No serious treatment-related adverse events were reported in the included RCTs. Reported events were predominantly transient and non-serious, including local post-treatment soreness, muscle soreness, immediate post-needling pain, mild post-exercise pain, and symptom exacerbation [37,39,43]. This pattern is broadly consistent with the adverse-event profile reported for other percutaneous needling interventions, such as dry needling, in which minor and transient adverse events are commonly described [65,66]. However, adverse-event definitions, reporting procedures, and ascertainment methods were not consistently described across trials, and absence of adverse-event reporting was not interpreted as evidence of zero events.

Importantly, this review was primarily designed to evaluate treatment effectiveness and was restricted to RCTs. Therefore, it was not designed to estimate the incidence of uncommon or serious adverse events, which may also be identified through observational studies, case series, or case reports. Accordingly, the available evidence does not allow a precise estimate of adverse-event incidence or firm conclusions regarding the overall safety profile of EL and MEL. Future RCTs should adopt standardized definitions, systematic surveillance, and complete reporting of adverse events to improve the quality and comparability of safety data across electrolysis protocols.

### 4.5. Treatment Parameters and Dose Considerations

Although considerable variability was observed across electrolysis protocols, several consistent treatment characteristics emerged among the included RCTs. For MEL, the most frequently investigated protocol consisted of ultrasound-guided application delivering approximately 25–30 mC per session, administered once weekly for four sessions and combined with therapeutic exercise [37,39,40,41,42]. Notably, most studies reporting favorable clinical outcomes converged on a similar delivered electrical charge, suggesting that the overall electrical dose may be more relevant than current intensity or application time considered independently. Accordingly, this regimen represents the most frequently investigated MEL dosing approach among the included RCTs; however, the available evidence is insufficient to establish it as an optimal dosing strategy. In contrast, conventional EL protocols demonstrated substantial variability in current intensity (1.5–6 mA), delivered electrical charge (72–810 mC), and treatment duration [38,44], precluding the identification of a standardized dosing regimen. Future adequately powered dose–response RCTs comparing different electrical charges, treatment frequencies, and numbers of treatment sessions are warranted to establish optimal treatment parameters.

### 4.6. Limitations

Several limitations should be considered when interpreting these findings. Although eight RCTs were included, the evidence base remains relatively small. Clinical and methodological heterogeneity was evident across studies, including variability in diagnostic terminology and criteria, electrolysis modality (EL or MEL), treatment parameters, co-interventions, comparator interventions, and follow-up duration. These differences limit direct comparisons across studies and may have contributed to variability in treatment effects. In addition, adverse-event reporting was inconsistent across studies, particularly regarding event definitions, severity, and surveillance methods, limiting the certainty of conclusions regarding safety. Several secondary outcomes were informed by only a limited number of studies, reducing the precision of some pooled estimates and precluding more detailed subgroup analyses. Most trials reported only short- to medium-term follow-up, limiting conclusions regarding long-term effects. Finally, publication bias could not be formally assessed because fewer than 10 studies were available for each meta-analysis.

## 5. Conclusions

This systematic review and meta-analysis suggests that electrolysis-based interventions, including EL and MEL, may improve pain intensity and shoulder-related disability in individuals with RC tendinopathy. Potential benefits were also observed for selected ROM outcomes, whereas evidence for PPT remains inconclusive. However, the certainty of the evidence ranged from low to moderate across outcomes, and the robustness of these findings is limited by the small number of RCTs, substantial clinical and methodological heterogeneity, and predominantly short- to medium-term follow-up. Safety evidence remains limited and imprecise due to the small number of studies and inconsistent adverse-event reporting. Although no serious treatment-related adverse events were reported, firm conclusions regarding the safety of EL and MEL cannot be drawn. Future adequately powered RCTs should use standardized diagnostic criteria, comparable background rehabilitation programs, direct EL-versus-MEL comparisons, and standardized adverse-event reporting, while also investigating dose–response relationships to clarify relative clinical effects and optimal treatment parameters.

## Figures and Tables

**Table 1:** Characteristics of included RCTs.

First Author (Year) Country	PEDro Score	Diagnosis	Participants (*n*) Mean Age (SD) Dropout (*n*)	Electrolysis Technique	Groups (*n*)	Outcomes (Instrument)	Assessment Instances	Results After Treatment	Between-Group Effect Estimates at Post-Treatment (95% CI)	Treatment-Related Adverse Events (*n*)	Study Conclusion	Source of Funding
**Arias-Buría (2015) [37] Spain**	7/10	SAPS with supraspinatus tendinopathy	36 (M 9, F 27) 57.0 (6.5) 0	MEL (EPTE^®^)	EG (17): MEL + TE (EE) CG (19): TE (EE)	(A) PI (NPRS) (B) Disability (DASH)	T0: baseline T1: during treatment (2 weeks) T2: post-treatment (4 weeks)	EG: ↓ PI * and ↓ disability * CG: ↓ PI * and ↓ disability * EG < CG: ↓ PI * and ↓ disability *	(A) PI: MD = 1.90 (95% CI 0.70 to 3.10). (B) DASH: MD = 9.50 (95% CI 1.90 to 17.20). Both favoring EG.	EG: 6/17 local post-treatment soreness; DOMS also reported. No serious treatment-related adverse events reported.	MEL combined with eccentric exercise improved short-term pain and disability compared with exercise alone, with clinically meaningful effects only for pain.	NR
**Kazemi (2016) [38] Spain**	5/10	Symptomatic infraspinatus tendon involvement	40 (M 20, F 20) 39.9 (3.9) 0	EL (EPI^®^)	EG 1 (10): EL (MTrPs) EG 2 (10): EL (Infraspinatus tendon) EG 3 (10): EL (MTrPs and infraspinatus tendon) CG: No treatment.	(A) PI (VAS) (B) Shoulder ROM (GNM)	T0: baseline T1: during treatment (1 week) T2: during treatment (2 weeks) T3: post-treatment (3 weeks)	EG 1: ↓ PI * and ↓ limited ROM * EG 2: ↓ PI * and ↓ limited ROM * EG 3: ↓ PI * and ↓ limited ROM * CG: ↓ PI and ↓ limited ROM EG3 < EG2 = EG1 < CG: ↓ PI * and ↓ limited ROM *	(A) PI: EG1 vs. CG: MD = −28.00 (95% CI −38.41 to −17.59); EG2 vs. CG: MD = −45.50 (95% CI −50.78 to −40.22); EG3 vs. CG: MD = −70.00 (95% CI −75.61 to −64.39). (B) ROM (restriction): ABD—EG1 vs. CG: MD = −24.00° (95% CI −26.65 to −21.35); EG2 vs. CG: −23.00° (−25.81 to −20.19); EG3 vs. CG: −43.00° (−45.81 to −40.19). IR—EG1 vs. CG: −15.50° (−26.65 to −4.35); EG2 vs. CG: −15.00° (−26.20 to −3.80); EG3 vs. CG: −40.00° (−51.26 to −28.74). ER—EG1 vs. CG: −21.00° (−24.77 to −17.23); EG2 vs. CG: −20.50° (−24.48 to −16.52); EG3 vs. CG: −50.00° (−52.92 to −47.08).	No harms or unintended effects reported; ascertainment method NR.	EL reduced pain and improved shoulder ROM, with greater benefits when applied to both infraspinatus MTrPs and tendon than to either site alone.	NR
**de Miguel Valtierra (2018) [39]** **Spain**	9/10	SAPS with supraspinatus tendon involvement	50 (M 23, F 27) 55 (12.4) 2	MEL (EPTE^®^)	EG (25): MEL + MT (joint mobilizations and soft tissue techniques) + TE CG (25): MT (joint mobilizations and soft tissue techniques) + TE	(A) PI (NPRS) (B) PPT (ALG) (C) Disability (DASH) (D) Disability (SPADI) (E) Self-reported improvement (GROC)	T0: baseline T1: post-treatment (5 weeks) T2: follow-up (12 weeks) T3: follow-up (24 weeks)	EG: ↓ PI *, ↓ disability * and ↑ PPT * CG: ↓ PI *, ↓ disability * and ↑ PPT * EG < CG: ↓ PI * and ↓ disability EG > CG: ↑ PPT	(A) PI: MD = −1.40 (95% CI −2.40 to −0.40). (B) PPT: C5–C6: MD = 0.00 (−0.50 to 0.50); deltoid: 0.10 (−0.70 to 0.90); 2nd metacarpal: −0.10 (−0.90 to 0.70); tibialis anterior: −0.40 (−1.40 to 0.60). (C) DASH: adj. MD = 1.70 (−0.30 to 3.70). (D) SPADI: MD = −12.50 (−18.90 to −6.10). PI and SPADI favoring EG; PPT and DASH showed no significant between-group differences.	EG: 6/25 participants reported muscle soreness. No serious treatment-related adverse events reported	MEL combined with manual therapy and exercise improved pain and function, but not disability or pressure pain sensitivity	No funding received
**Rodríguez-Huguet (2020) [40] Spain**	7/10	Supraspinatus tendinopathy	36 (M 27, F 9) 43 (9.9) 0	MEL (EPTE^®^)	EG (18): MEL + TE (EE) CG (18): MTrPs dry needling + TE (EE)	(A) PI (NPRS) (B) PPT (ALG) (C) Shoulder ROM (GNM)	T0: baseline T1: post-treatment (4 weeks) T2: follow-up (1 month) T3: follow-up (1 year)	EG: ↓ PI *, ↑ PPT * and ↑ ROM * CG: ↓ PI *, ↑ PPT * and ↑ ROM * EG < CG: ↓ PI * EG > CG: ↑ PPT * and ↑ ROM *	A) PI: MD = −1.99 (95% CI −3.44 to −0.55). (B) PPT: proximal: MD = 1.31 (0.67 to 1.96); middle: 1.23 (0.58 to 1.87); distal: 1.00 (0.32 to 1.68). (C) ROM: FLEX: MD = 10.49° (−1.98 to 22.95); EXT: 7.25° (3.77 to 10.74); ER: 19.27° (12.64 to 25.90). All favoring EG; FLEX was not statistically significant.	No adverse events reported; ascertainment method NR.	MEL was more effective than trigger point dry needling for reducing pain and improving shoulder ROM and pressure pain thresholds in patients with supraspinatus tendinopathy	No funding received
**Góngora-Rodríguez (2024) [41]** **Spain**	5/10	Supraspinatus tendinopathy	50 (M 36, F 14) 44.2 (11.8) 0	MEL (EPTE^®^)	EG (25): MEL + PENS + TE (EE) CG (25): TENS + US + TE (EE)	(A) PI (NPRS) (B) Shoulder strength (DYN) (C) Tendon morphology (USG) (D) Disability (DASH) (E) Disability (SPADI) (F) Muscle activity (EMG)	T0: baseline T1: post-treatment (4 weeks) T2: follow-up (12 weeks) T3: follow-up (24 weeks)	EG: ↓ PI *, ↓ Disability *, ↑ Strength *, ↑ Muscle activity *, and ↑ Tendon morphology * CG: ↓ PI *, ↓ Disability *, ↑ Strength *, ↑ Muscle activity *, and ↑ Tendon morphology * EG < CG: ↓ PI * and ↓ Disability * EG > CG: ↑ Strength *, ↑ Muscle activity *, and ↑ Tendon morphology *	(A) PI: MD = −2.96 (95% CI −4.06 to −1.86). (B) Shoulder strength: ABD: MD = 0.74 (0.37 to 1.11); IR: 0.32 (0.03 to 0.61); ER: 0.58 (0.27 to 0.89). (C) Tendon morphology (USG): hypervascularization: RR = 0.57 (0.38 to 0.84). (D) DASH: MD = −33.16 (−42.07 to −24.25). (E) SPADI: MD = −30.96 (−39.69 to −22.23). All favoring EG.	No adverse events reported; ascertainment method NR.	MEL combined with PENS and eccentric exercise improved pain, function, strength, muscle activity, and tendon morphology in supraspinatus tendinopathy.	No funding received
**Góngora-Rodríguez (2025) [42]** **Spain**	7/10	Supraspinatus tendinopathy	46 (M 32, F 14) 44.2 (12.3) 0	MEL (EPTE^®^)	EG (23): MEL + PENS + TE (EE) CG (23): TENS + US + TE (EE)	(A) PI (NPRS) (B) PPT (ALG) (C) Shoulder ROM (GNM) (D) Disability (DASH) (E) Disability (SPADI)	T0: baseline T1: post-treatment (4 weeks) T2: follow-up (12 weeks) T3: follow-up (24 weeks)	EG: ↓ PI *, ↑ PPT *, ↑ ROM *, and ↓ Disability * CG: ↓ PI *, ↑ PPT *, ↑ ROM *, and ↓ Disability * EG < CG: ↓ PI * and ↓ Disability * EG > CG: ↑ PPT * and ↑ ROM *	(A) PI: MD = −3.61 (95% CI −4.53 to −2.69). (B) PPT: proximal: MD = 0.88 (0.48 to 1.28); middle: 0.91 (0.48 to 1.34); distal: 0.89 (0.38 to 1.40). (C) ROM: FLEX: MD = 22.70° (13.35 to 32.05); EXT: 4.04° (0.83 to 7.25); ABD: 14.26° (4.87 to 23.65); IR: 16.83° (7.60 to 26.06); ER: 12.70° (3.85 to 21.55). (D) DASH: MD = −36.83 (−45.48 to −28.18). (E) SPADI: MD = −35.35 (−43.43 to −27.27). All favoring EG.	No adverse events reported; ascertainment method NR.	MEL combined with PENS and eccentric exercise produced superior clinical outcomes compared with conventional electrotherapy and exercise in patients with supraspinatus tendinopathy.	No funding received
**Magalhães (2025) [43]** **Brazil**	8/10	Chronic imaging-confirmed RC tendinopathy	42 (M 6, F 36) 53.0 (9.6) 2	MEL (EPTE^®^)	EG (21): MEL + TE (GRASP) CG (21): TE (GRASP)	(A) PI (NPRS) (B) Disability (SPADI) (C) Global perceived effect (GPE) (D) Adverse events (E) Exercise adherence	T0: baseline T1: post-treatment (8 weeks)	EG: ↓ PI * and ↓ Disability * CG: ↓ PI * and ↓ Disability * EG = CG: PI and Disability	(A) PI: MD = −1.23 (95% CI −2.54 to 0.07). (B) SPADI: MD = 7.17 (95% CI −7.02 to 21.35). PI favored EG and SPADI favored CG; neither difference was statistically significant.	EG: 12/21; immediate post-needling pain (5), pain ≤24 h (4), mild post-exercise pain (2), symptom exacerbation (1). CG: 14/21; mild post-exercise muscle soreness (13), symptom exacerbation (1).	MEL plus therapeutic exercise was not superior to therapeutic exercise alone for pain or disability in RC tendinopathy.	No funding received
**Kużdżał (2026) [44]** **Poland**	8/10	Subscapularis tendinopathy	30 (M 20, F 10) 45.8 (12.9) 0	EL (EPI^®^)	EG (15): EL + Physical agents (ESWT) + MT + TE CG (15): Physical agents (ESWT) + MT + TE	(A) PI (VAS) (B) Disability (DASH) (C) Tendon stiffness (MyotonPRO) (D) PPT (ALG) (E) Internal rotation ROM (GNM) (F) Internal rotation strength (DYN) (G) Tendon morphology (USG) (H) Tissue perfusion (LDF) (I) Tenascin-C (ELISA) (J) Subscapularis clinical tests (Lift-off, Belly-press, Bear-hug)	T0: baseline (pre-treatment) T1: post-treatment (8 weeks)	EG: ↓ PI *, ↓ Disability *, ↓ Tendon stiffness *, ↑ PPT *, ↑ ROM *, ↑ Tissue perfusion *, ↑ Tenascin-C * and ↑ Strength * CG: ↓ PI *, ↓ Disability *, ↓ Tendon stiffness *, ↑ PPT *, ↑ ROM *, ↑ Tissue perfusion *, ↑ Tenascin-C * and ↑ Strength * EG < CG: ↓ Disability *, ↓ Tendon stiffness*, and ↑ PPT * EG > CG: ↑ ROM *, ↑ Tenascin-C * and ↑ Strength * EG = CG: ↓ PI and ↑ Tissue perfusion	(A) PI: MD = 0.07 (95% CI −0.64 to 0.77). (B) DASH: MD = −3.91 (−5.84 to −1.97). (C) Tendon stiffness: MD = −31.29 N/m (−36.65 to −25.93). (D) PPT: MD = 10.01 kPa (9.07 to 10.94). (E) IR ROM: MD = 5.29° (4.18 to 6.39). (F) IR strength: MD = 1.53 kgF (1.15 to 1.92). All favoring EG, except PI, which showed no significant between-group difference.	No treatment-related adverse events reported; ascertainment method NR.	EL plus multimodal rehabilitation improved disability, tendon properties, ROM, strength, and PPT, but not pain, versus multimodal rehabilitation alone.	No funding received

**Abbreviations:** ABD, abduction; ALG, algometry; CG, control group; CI, confidence interval; DASH, Disabilities of the Arm, Shoulder and Hand; DOMS, delayed-onset muscle soreness; DYN, handheld dynamometry; EE, eccentric exercise; EG, experimental group; EG1–EG3, experimental groups 1–3; EL, electrolysis; ELISA, enzyme-linked immunosorbent assay; EMG, electromyography; EPI^®^, registered trademark of the Intratissue Percutaneous Electrolysis System; EPTE^®^, registered trademark of the Therapeutic Percutaneous Electrolysis System; ER, external rotation; ESWT, extracorporeal shock wave therapy; EXT, extension; F, female; FLEX, flexion; GNM, goniometry; GRASP, Getting it Right: Addressing Shoulder Pain; GROC, Global Rating of Change; IR, internal rotation; LDF, laser Doppler flowmetry; M, male; MD, mean difference; MEL, microelectrolysis; MEP^®^, registered trademark of the Percutaneous Microelectrolysis System; MRI, magnetic resonance imaging; MTrPs, myofascial trigger points; MT, manual therapy; NPRS, Numeric Pain Rating Scale; NR, not reported; PENS, percutaneous electrical nerve stimulation; GPE, global perceived effect; PI, pain intensity; PPT, pressure pain threshold; RC, rotator cuff; ROM, range of motion; SAPS, subacromial pain syndrome; SD, standard deviation; SPADI, Shoulder Pain and Disability Index; TE, therapeutic exercise; TENS, transcutaneous electrical nerve stimulation; US, therapeutic ultrasound; USG, ultrasound imaging; VAS, visual analogue scale; ↑, increase; ↓, decrease; *, *p* < 0.05.

**Table 2:** Technical parameters of the electrolysis modalities included in the RCTs.

Electrolysis Parameters	Arias-Buría et al. (2015) [37]	Kazemi et al. (2016) [38]	de Miguel Valtierra et al. (2018) [39]	Rodríguez-Huguet et al. (2020) [40]	Góngora-Rodríguez et al. (2024) [41]	Góngora-Rodríguez et al. (2025) [42]	Magalhães et al. (2025) [43]	Kużdżał et al. (2026) [44]
Electrolysis modality	Microelectrolysis	Electrolysis	Microelectrolysis	Microelectrolysis	Microelectrolysis	Microelectrolysis	Microelectrolysis	Electrolysis
Device	EPTE^®^ V01 (Ionclinics, Valencia, Spain)	EPI^®^ EEC Cesmar (Valencia, Spain)	EPTE^®^ V01 (Ionclinics, Valencia, Spain)	EPTE^®^ (Ionclinics & Deionic S.L., Valencia, Spain)	EPTE^®^ Bipolar System (Ionclinics & Deionics S.L., Valencia, Spain)	EPTE^®^ Bipolar System (Ionclinics & Deionics S.L., Valencia, Spain)	STRIAT, Model S19 (IBRAMED, Amparo, Brazil)	AS Super 4D Digital (AS Albrecht GmbH, Bernau am Chiemsee, Germany)
Ultrasound-guided	Yes	Yes	Yes	Yes	Yes	Yes	No	Yes
Intensity (µA and mA)	350 µA (0.35 mA)	6000 µA (6 mA)	350 µA (0.35 mA)	350 µA (0.35 mA)	350 µA (0.35 mA)	350 µA (0.35 mA)	900 µA (0.9 mA)	1500 µA (1.5 mA)
Application time (seconds)	72 s	4 s/application (3 applications)	90 s	72 s	72 s	72 s	40 s	180 s/application (3 applications)
Needle size	0.30 × 25 mm	Size according to subject anthropometry	0.30 × 25 mm	Not reported	0.30 × 40 mm	0.30 × 40 mm	0.25 × 30 mm	0.30 × 30 mm
Delivered charge per session	25.2 mC	72 mC	31.5 mC	25.2 mC	25.2 mC	25.2 mC	36 mC	810 mC
Treatment frequency	1 s/week	1 s/week	1 s/week	1 s/week	1 s/week	1 s/week	1 s/week	1 s/week
Total sessions	4	3	5	4	4	4	6	8
Intervention protocol	Microelectrolysis targeting the supraspinatus tendon, combined with eccentric exercise.	Electrolysis applied to the infraspinatus muscle–tendon complex, targeting trigger points, tendon pain, or both.	Microelectrolysis targeting the supraspinatus tendon, combined with exercise and manual therapy.	Microelectrolysis targeting the supraspinatus tendon, combined with eccentric exercise.	Microelectrolysis targeting the supraspinatus tendon, combined with PENS and eccentric exercise.	Microelectrolysis targeting the supraspinatus tendon, combined with PENS and eccentric exercise.	Microelectrolysis applied to the rotator cuff tendon insertion region, combined with therapeutic exercise.	Electrolysis targeting the subscapularis tendon, combined with ECSWT, manual therapy, and exercise.

**Abbreviations:** AS, Albrecht System; ECSWT, Extracorporeal Shockwave Therapy; EEC, Electrolysis Equipment Cesmar; EPI^®^, Intratissue Percutaneous Electrolysis; EPTE^®^, Therapeutic Percutaneous Electrolysis; mA, milliampere; mC, millicoulomb; mm, millimeter; PENS, percutaneous peripheral nerve stimulation; s, second; µA, microampere.

**Table 3:** Summary of findings and certainty of the evidence (GRADE) for EL and MEL compared with control interventions in RC tendinopathy.

Outcome		Certainty Assessment							№ of Patients		Effect Estimate (95% CI)	Certainty	Importance
Outcome Category	Comparison/Sub-Outcome	Studies	Study Design	Risk of Bias	Inconsistency	Indirectness	Imprecision	Publication Bias	EL/MEL Group	Control Group			
**Pain intensity**	Post-treatment (NPRS and VAS): MEL plus TE vs. TE	3 [37,40,43]	RCTs	not serious ^a^ (0)	serious ^b^ (−1)	not serious ^c^ (0)	serious ^d^ (−1)	Not assessed ^e^	56	58	SMD = −0.4 (−1.2 to 0.5)	⨁⨁◯◯ Low	CRITICAL
	Post-treatment (NPRS and VAS): MEL plus MPT vs. MPT	3 [39,41,42]	RCTs	not serious ^a^ (0)	serious ^b^ (−1)	not serious ^c^ (0)	serious ^d^ (−1)	Not assessed ^e^	73	73	SMD = −1.4 (−2.1 to −0.6)	⨁⨁◯◯ Low	CRITICAL
	3-month follow-up (NPRS): MEL plus MPT vs. MPT	2 [39,42]	RCTs	not serious ^a^ (0)	not serious ^b^ (0)	not serious ^c^ (0)	serious ^d^ (−1)	Not assessed ^e^	48	48	MD = 2.8 points lower (2.0 to 3.7 lower)	⨁⨁⨁◯ Moderate	CRITICAL
	6-month follow-up (NPRS): MEL plus MPT vs. MPT	2 [39,42]	RCTs	not serious ^a^ (0)	not serious ^b^ (0)	not serious ^c^ (0)	serious ^d^ (−1)	Not assessed ^e^	48	48	MD = 2.9 points lower (2.1 to 3.7)	⨁⨁⨁◯ Moderate	CRITICAL
	Post-treatment (VAS and NPRS): Overall	8 [37,38,39,40,41,42,43,44]	RCTs	not serious ^a^ (0)	serious ^b^ (−1)	not serious ^c^ (0)	serious ^d^ (−1)	Not assessed ^e^	174	156	SMD = −1.44 (−2.2 to −0.7)	⨁⨁◯◯ Low	CRITICAL
	First follow-up within the first 3 months (NPRS): Overall	3 [39,40,42]	RCTs	not serious ^a^ (0)	serious ^b^ (−1)	not serious ^c^ (0)	serious ^d^ (−1)	Not assessed ^e^	66	66	MD = 2.25 points lower (1.1 to 3.4)	⨁⨁◯◯ Low	CRITICAL
	6-month follow-up (NPRS): Overall	2 [39,42]	RCTs	not serious ^a^ (0)	not serious ^b^ (0)	not serious ^c^ (0)	serious ^d^ (−1)	Not assessed ^e^	48	48	MD = 2.89 points lower (2.1 to 3.7)	⨁⨁⨁◯ Moderate	CRITICAL
**Disability**	Post-treatment (DASH and SPADI): MEL plus TE vs. TE	2 [37,43]	RCTs	not serious ^a^ (0)	serious ^b^ (−1)	not serious ^c^ (0)	serious ^d^ (−1)	Not assessed ^e^	38	40	SMD = −0.4 (−1.9 to 1.0)	⨁⨁◯◯ Low	CRITICAL
	Post-treatment (DASH and SPADI): MEL plus MPT vs. MPT	3 [39,41,42]	RCTs	not serious ^a^ (0)	not serious ^b^ (0)	not serious ^c^ (0)	serious ^d^ (−1)	Not assessed ^e^	73	73	SMD = −1.0 (−1.3 to −0.7)	⨁⨁⨁◯ Moderate	CRITICAL
	3-month follow-up (SPADI): MEL plus MPT vs. MPT	2 [39,42]	RCTs	not serious ^a^ (0)	serious ^b^ (−1)	not serious ^c^ (0)	serious ^d^ (−1)	Not assessed ^e^	48	48	MD = 25.9 points lower (8.2 to 43.7)	⨁⨁◯◯ Low	CRITICAL
	6-month follow-up (SPADI): MEL plus MPT vs. MPT	2 [39,42]	RCTs	not serious ^a^ (0)	serious ^b^ (−1)	not serious ^c^ (0)	serious ^d^ (−1)	Not assessed ^e^	48	48	MD = 12.4 points lower (2.1 to 22.6)	⨁⨁◯◯ Low	CRITICAL
	Post-treatment (DASH and SPADI): Overall	6 [37,39,41,42,43,44]	RCTs	not serious ^a^ (0)	serious ^b^ (−1)	not serious ^c^ (0)	serious ^d^ (−1)	Not assessed ^e^	126	128	SMD = −0.81 (−1.3 to −0.4)	⨁⨁◯◯ Low	CRITICAL
	3-month follow-up (SPADI): Overall	2 [39,42]	RCTs	not serious ^a^ (0)	serious ^b^ (−1)	not serious ^c^ (0)	serious ^d^ (−1)	Not assessed ^e^	48	48	MD = −25.92 (−43.65 to −8.19)	⨁⨁◯◯ Low	CRITICAL
	6-month follow-up (SPADI): Overall	2 [39,42]	RCTs	not serious ^a^ (0)	serious ^b^ (−1)	not serious ^c^ (0)	serious ^d^ (−1)	Not assessed ^e^	48	48	MD = −12.36 (−22.64 to −2.07)	⨁⨁◯◯ Low	CRITICAL
**ROM**	Abduction—post-treatment (goniometry): Overall	3 [38,40,42]	RCTs	not serious ^a^ (0)	serious ^b^ (−1)	not serious ^c^ (0)	serious ^d^ (−1)	Not assessed ^e^	71	51	MD = 26.05° greater (16.34 to 35.76)	⨁⨁◯◯ Low	IMPORTANT
	Internal rotation—post-treatment (goniometry): Overall	4 [38,40,42,44]	RCTs	not serious ^a^ (0)	serious ^b^ (−1)	not serious ^c^ (0)	serious ^d^ (−1)	Not assessed ^e^	86	66	MD = 17.4° greater (8.4 to 26.3)	⨁⨁◯◯ Low	IMPORTANT
	Flexion—post-treatment (goniometry): Overall	2 [40,42]	RCTs	not serious ^a^ (0)	not serious ^b^ (0)	not serious ^c^ (0)	serious ^d^ (−1)	Not assessed ^e^	41	41	MD = 12.51° greater (4.4 to 20.6)	⨁⨁⨁◯ Moderate	IMPORTANT

**Abbreviations:** CI—confidence interval; DASH—Disabilities of the Arm, Shoulder and Hand; EL—electrolysis; GRADE—Grading of Recommendations Assessment, Development and Evaluation; MD—mean difference; MEL—microelectrolysis; MPT—multimodal physiotherapy; NPRS—Numeric Pain Rating Scale; PPT—pressure pain threshold; RCTs—randomized controlled trials; ROM—range of motion; SMD—standardized mean difference; SPADI—Shoulder Pain and Disability Index; TE—therapeutic exercise; VAS—Visual Analog Scale. **Explanations:** a. Not downgraded. Across all included RCTs, overall RoB was low in 4/8 studies (50.0%), some concerns in 3/8 (37.5%), and high in 1/8 (12.5%). For the overall post-treatment pain analysis, sensitivity analysis excluding the single study at high overall RoB did not materially alter the direction or statistical significance of the pooled effect; b. Certainty was downgraded for inconsistency when substantial heterogeneity was observed across studies (I^2^ > 50%); c. No downgrade was applied for indirectness, which was judged according to the relevance of the population, intervention, comparator, and outcomes (PICO) to the review question; d. Imprecision was judged considering CI width, whether intervals crossed the null or clinically important thresholds, and the number of participants or events contributing to the outcome (<400 participants); e. Publication bias was not formally examined because fewer than 10 studies contributed to the analyses; f. Outcome importance was established a priori based on GRADE guidance and its clinical relevance for decision-making. GRADE certainty of evidence: ⨁⨁⨁⨁, high; ⨁⨁⨁◯, moderate; ⨁⨁◯◯, low; ⨁◯◯◯, very low.

## Data Availability

The data supporting the findings of this study are available within the article and its Supplementary Materials. Additional data are available from the corresponding author upon reasonable request.

## Supplementary Materials

The following supporting information can be downloaded at: https://www.mdpi.com/article/10.3390/jcm15207983/s1, Table S1, presenting the complete search strategy for all electronic databases; Table S2, summarizing the study-level data used in the quantitative and narrative syntheses; Table S3, detailing the diagnostic and eligibility criteria of the included RCTs; Table S4, reporting the methodological quality assessment of the included studies using the Physiotherapy Evidence Database (PEDro) scale; Supplementary Material File A, PRISMA 2020 Checklist can be found at this link: https://drive.google.com/file/d/1Oddmr81Eq6ITeUab6MxYCxjQv84NH8Pl/view?usp=sharing (accessed on 14 September 2026).

## Abbreviations

The following abbreviations are used in this manuscript:

CI	Confidence Interval
EL	Percutaneous Electrolysis
GRADE	Grading of Recommendations Assessment, Development and Evaluation
I^2^	Higgins’ Inconsistency Statistic
mA	Milliampere
MCID	Minimal Clinically Important Difference
MD	Mean Difference
MeSH	Medical Subject Headings
MEL	Microelectrolysis
PICOS	Population, Intervention, Comparator, Outcomes, and Study Design
PPT	Pressure Pain Threshold
PRISMA	Preferred Reporting Items for Systematic Reviews and Meta-Analyses
PROSPERO	International Prospective Register of Systematic Reviews
RC	Rotator Cuff
RCT	Randomized Controlled Trial
REML	Restricted Maximum Likelihood
RoB	Risk of Bias
RoB 2	Risk of Bias 2
ROM	Range of Motion
SAIS	Subacromial Impingement Syndrome
SAPS	Subacromial Pain Syndrome
SD	Standard Deviation
SE	Standard Error
SMD	Standardized Mean Difference
μA	Microampere
