# Supplementary material for: Percutaneous Electrolysis and Microelectrolysis for Rotator Cuff Tendinopathy: A Systematic Review and Meta-Analysis of Randomized Controlled Trials
Source: J Clin Med. 2026 Oct 15;15(20):7983. doi: 10.3390/jcm15207983 (PMC13706561; doi:10.3390/jcm15207983)
Supplement: Supplementary file 1 [file jcm-15-07983-s001.zip › jcm-4485763-supplementary.pdf]

Supplementary Table S1. Search strategy (last updated September 14, 2026).

Search		Identification of studies via databases and registers									Identification of studies via other methods
	**Keywords**	PubMed	Scopus	Web of Science	CINAHL	MEDLINE	ScienceDirect	Cochrane Library	PEDro	**TOTAL**	Google Scholar
**s1**	*″Electrolysis″*	15.007	324.234	73.953	227	14.666	175.913	164		589.498	
**s2**	*″Electroacupuncture″*	9.587	31.64	9.062	2.445	8.992	6.940	4.541		32.575	
**s3**	*″Direct current″*	19.567	164.691	58.123	3.130	19.288	141.239	9.003		415.041	
**s4**	*″Intratissue percutaneous electrolysis″*	14	91	21	11	13	13	7		170	
**s5**	*″Microelectrolysis″*	257	2.017	269	4	70	570	12		3.199	
**s6**	*S1 OR S2 OR S3 OR S4 OR S5*	44.009	445.757	140.540	5.784	42.804	311.862	13.695		961.647	
**s7**	*″Musculoskeletal Pain″*	14.169	75.410	16.737	6.086	13.949	27.273	3.696		157.320	
**s8**	*″Tendinopathy″*	11.689	25.647	9.424	5.727	11.535	13.517	2.011		79.550	
**s9**	*″Rotator Cuff″*	22.341	51.820	21.609	9.647	22.039	31.991	3.737		163.184	
**s10**	*″Shoulder Pain″*	14.167	44.810	11.940	7.725	13.991	25.815	5.770		124.218	
**s11**	*″Shoulder Impingement Syndrome″*	2.379	6.627	833	1.668	2.366	1.212	998		13.717	
**s12**	*S7 OR S8 OR S9 OR S10 OR S11*	56.789	167.143	54.242	27.133	56.047	81.789	13.558		456.701	
**s13**	S6 AND S12	182	2.072	232	78	170	88	197	10	3.029	151

*Search algorithm used for formal databases: *(″Electrolysis″ OR ″Electroacupuncture″ OR ″Direct Current″ OR ″Intratissue Percutaneous Electrolysis″ OR ″Microelectrolysis″) AND (″Musculoskeletal Pain″ OR ″Tendinopathy″ OR ″Rotator Cuff″ OR ″Shoulder Pain″ OR ″Shoulder Impingement Syndrome″)* **Search algorithm for other identification of studies via other methods: *(″Electrolysis″ OR ″Intratissue Percutaneous Electrolysis″ OR ″Microelectrolysis″) AND (“Rotator Cuff″ OR ″Shoulder Pain″ OR ″Shoulder Impingement Syndrome″).*

Supplementary Table S2. Summary of Study-Level Data Extracted for Quantitative and Narrative Synthesis.

Study	Country	Diagnostic criteria	Electrolysis modality / target tissue	Intervention	Comparator / background rehabilitation	Ultrasound guidance	Dose per session	Sessions	Outcome / instrument	Assessment time point	Final synthesis	Experimental group n	mean	SD	Comparator group n	mean	SD	Effect measure	Effect estimate	95% CI	Effect estimate source	Data handling / notes	Adverse events	Funding
**Arias-Buría et al. (2015)**	Spain	Chronic unilateral subacromial pain syndrome (≥3 months), NPRS ≥4/10, positive impingement signs, MRI-confirmed supraspinatus tendinopathy.	MEL (EPTE®); supraspinatus tendon	MEL + eccentric therapeutic exercise	Eccentric therapeutic exercise	Yes	350 µA × 72 s = 25.2 mC/session	4; 1/week	Pain intensity (NPRS, 0–10)	Post-treatment	Meta-analysis	17	1.40	1.20	19	3.10	2.10	Hedges g	-0.96	-1.64 to -0.28	Calculated from group-level data used in the review synthesis	Original RCT also reported between-group difference in change = 1.9 (95% CI 0.7 to 3.1).	EG: 6/17 local post-treatment soreness; DOMS also reported; no serious treatment-related events.	Not reported
**Kazemi et al. (2016)**	Spain	Infraspinatus trigger points and tendon pain ≥3 months; VAS ≥65/100; pain-limited abduction, internal rotation, and external rotation.	EL (EPI®); infraspinatus trigger points, tendon, or both	Three EL groups: trigger points; tendon; trigger points + tendon	No treatment (shared control)	Yes	6 mA; 4 s/application × 3 = 72 mC/session	3; 1/week	Pain intensity (VAS, converted to 0–10)	Post-treatment	Meta-analysis	10	2.40	0.20	3	4.10	0.70	Hedges g	-4.53	-6.77 to -2.29	Calculated from group-level data used in the review synthesis	EL – trigger points vs no treatment; shared control split for meta-analysis. Original control n=10; analysis n split 3/3/4 to avoid double-	No harms or unintended effects reported.	Not reported

																						counting.		
**Kazemi et al. (2016)**	Spain	Infraspinatus trigger points and tendon pain ≥3 months; VAS ≥65/100; pain-limited abduction, internal rotation, and external rotation.	EL (EPI®); infraspinatus trigger points, tendon, or both	Three EL groups: trigger points; tendon; trigger points + tendon	No treatment (shared control)	Yes	6 mA; 4 s/application × 3 = 72 mC/session	3; 1/week	Pain intensity (VAS, converted to 0–10)	Post-treatment	Meta-analysis	10	1.20	0.50	3	4.10	0.70	Hedges g	-4.98	-7.38 to -2.58	Calculated from group-level data used in the review synthesis	EL – tendon vs no treatment; shared control split for meta-analysis. Original control n=10; analysis n split 3/3/4 to avoid double-counting.	No harms or unintended effects reported.	Not reported
**Kazemi et al. (2016)**	Spain	Infraspinatus trigger points and tendon pain ≥3 months; VAS ≥65/100; pain-limited abduction, internal rotation, and external rotation.	EL (EPI®); infraspinatus trigger points, tendon, or both	Three EL groups: trigger points; tendon; trigger points + tendon	No treatment (shared control)	Yes	6 mA; 4 s/application × 3 = 72 mC/session	3; 1/week	Pain intensity (VAS, converted to 0–10)	Post-treatment	Meta-analysis	10	3.00	0.40	4	4.10	0.70	Hedges g	-2.09	-3.46 to -0.72	Calculated from group-level data used in the review synthesis	EL – trigger points + tendon vs no treatment; shared control split for meta-analysis. Original control n=10; analysi	No harms or unintended effects reported.	Not reported

																						s n split 3/3/4 to avoid double-counting.		
**de Miguel Valtierra et al. (2018)**	Spain	Unilateral shoulder pain ≥3 months; NPRS ≥4/10 during arm elevation; positive painful arc and ≥1 positive impingement/rotator cuff test.	MEL (EPTE®); supraspinatus tendon	MEL + manual therapy + therapeutic exercise	Same manual therapy + therapeutic exercise	Yes	350 µA × 90 s = 31.5 mC/session	5; 1/week	Pain intensity (NPRS, 0–10)	Post-treatment	Meta-analysis	25	3.50	1.40	25	4.80	2.40	Hedges g	-0.65	-1.21 to -0.09	Calculated from group-level data used in the review synthesis	Original RCT reported between-group difference in change = −1.4 (95% CI −2.4 to −0.4).	EG: 6/25 muscle soreness; no serious treatment-related events.	No funding received
**Rodríguez-Huguet et al. (2020)**	Spain	Ultrasound-confirmed supraspinatus tendinopathy; age 25–60 years; positive Hawkins–Kennedy and painful arc; active pain and tendon hyperalgesia.	MEL (EPTE®); supraspinatus tendon	MEL + eccentric therapeutic exercise	Trigger-point dry needling + same eccentric exercise	Yes	350 µA × 72 s = 25.2 mC/session	4; 1/week	Pain intensity (NPRS, 0–10)	Post-treatment	Meta-analysis	18	2.70	2.60	18	4.10	1.40	Hedges g	-0.66	-1.31 to 0.00	Calculated from group-level data used in the review synthesis	Original RCT reported adjusted between-group difference = −1.99 (95% CI −3.44 to −0.55).	No adverse events reported.	No funding received
**Góngora-Rodríguez et al. (2024)**	Spain	Adults with symptomatic supraspinatus tendinopathy; ultrasound-confirmed tendon changes; pain	MEL (EPTE®); supraspinatus tendon	MEL + PENS + eccentric therapeutic exercise	TENS + therapeutic ultrasound + same eccentric exercise	Yes	350 µA × 72 s = 25.2 mC/session	4; 1/week	Pain intensity (NPRS, 0–10)	Post-treatment	Meta-analysis	25	2.10	1.30	25	4.40	1.40	Hedges g	-1.68	-2.32 to -1.04	Calculated from group-level data used in the review	Original RCT reported between-group change score =	No adverse events reported.	No funding received

		with resisted movement; positive Jobe, Neer, Hawkins–Kennedy.																			w synthesis	−2.96; 95% CI not reported.		
**Góngora-Rodríguez et al. (2025)**	Spain	Adults 18–65 years with supraspinatus tendinopathy; pain at tendon insertion/resisted movement/palpation; positive Neer, Jobe, Hawkins–Kennedy.	MEL (EPTE®); supraspinatus tendon	MEL + PENS + eccentric therapeutic exercise	TENS + therapeutic ultrasound + same eccentric exercise	Yes	350 µA × 72 s = 25.2 mC/session	4; 1/week	Pain intensity (NPRS, 0–10)	Post-treatment	Meta-analysis	23	1.60	1.70	23	4.60	1.60	Hedges g	-1.79	-2.47 to -1.11	Calculated from group-level data used in the review synthesis	Original RCT reported between-group change score = −3.61; 95% CI not reported.	No adverse events reported.	No funding received
**Magalhães et al. (2025)**	Brazil	Adults with chronic rotator cuff tendinopathy and shoulder pain ≥3 months.	MEL (MEP/IPM); rotator cuff tendon insertion region	MEL + therapeutic exercise	Same therapeutic exercise program	No	900 µA × 40 s = 36 mC/session	6; 1/week	Pain intensity (NPRS, 0–10)	8 weeks	Meta-analysis	21	4.40	2.70	21	3.20	2.60	Hedges g	0.44	-0.16 to 1.05	Calculated from group-level data used in the review synthesis	Original RCT reported mixed-model between-group difference = −1.23 (95% CI −2.54 to 0.07).	EG: 12/21 nonserious events; CG: 14/21 nonserious expected events; no unexpected serious adverse effects.	No funding received
**Kuźdżał et al. (2026)**	Poland	Imaging-confirmed subscapularis tendinopathy; age 25–65 years; shoulder pain ≥1 month; VAS	EL (PNE/EPI); subscapularis tendon	EL + ESWT + manual therapy + exercise	Same ESWT + manual therapy + exercise	Yes	1.5 mA × 180 s × 3 = 810 mC/session	8; 1/week	Pain intensity (VAS)	8 weeks	Meta-analysis	15	0.60	0.50	15	1.20	0.40	Hedges g	-1.29	-2.06 to -0.52	Calculated from group-level data used in the review	Original RCT reported between-group difference in	No treatment-related adverse events reported.	No funding received

		≥4/10; positive subscapularis test.																			w synthesis	change = 0.07 (95% CI −0.64 to 0.77).		
**de Miguel Valtierra et al. (2018)**	Spain	Unilateral shoulder pain ≥3 months; NPRS ≥4/10 during arm elevation; positive painful arc and ≥1 positive impingement/rotator cuff test.	MEL (EPTE®); supraspinatus tendon	MEL + manual therapy + therapeutic exercise	Same manual therapy + therapeutic exercise	Yes	350 µA × 90 s = 31.5 mC/session	5; 1/week	Pain intensity (NPRS, 0–10)	3 months	Meta-analysis	25	2.20	2.00	25	4.90	2.80	MD	-2.70	-4.05 to -1.35	Calculated from group-level data used in the review synthesis	Original RCT reported between-group difference in change = −2.6 (95% CI −4.0 to −1.5).	EG: 6/25 muscle soreness; no serious treatment-related events.	No funding received
**Rodríguez-Huguet et al. (2020)**	Spain	Ultrasound-confirmed supraspinatus tendinopathy; age 25–60 years; positive Hawkins–Kennedy and painful arc; active pain and tendon hyperalgesia.	MEL (EPTE®); supraspinatus tendon	MEL + eccentric therapeutic exercise	Trigger-point dry needling + same eccentric exercise	Yes	350 µA × 72 s = 25.2 mC/session	4; 1/week	Pain intensity (NPRS, 0–10)	1 month (source-reported)	Meta-analysis	18	1.80	2.40	18	2.80	1.70	MD	-1.00	-2.36 to 0.36	Calculated from group-level data used in the review synthesis	This source time point has previously been grouped in the review's short-term follow-up analysis; Original RCT reported adjusted between-group	No adverse events reported.	No funding received

																						difference = −1.61 (95% CI −3.03 to −0.18).		
**Góngora-Rodríguez et al. (2025)**	Spain	Adults 18–65 years with supraspinatus tendinopathy; pain at tendon insertion/resisted movement/palpation; positive Neer, Jobe, Hawkins–Kennedy.	MEL (EPTE®); supraspinatus tendon	MEL + PENS + eccentric therapeutic exercise	TENS + therapeutic ultrasound + same eccentric exercise	Yes	350 µA × 72 s = 25.2 mC/session	4; 1/week	Pain intensity (NPRS, 0–10)	12 weeks	Meta-analysis	23	0.80	1.20	23	3.70	2.30	MD	-2.90	-3.96 to -1.84	Calculated from group-level data used in the review synthesis	Original RCT reported between-group change score = −3.41; 95% CI not reported.	No adverse events reported.	No funding received
**de Miguel Valtierra et al. (2018)**	Spain	Unilateral shoulder pain ≥3 months; NPRS ≥4/10 during arm elevation; positive painful arc and ≥1 positive impingement/rotator cuff test.	MEL (EPTE®); supraspinatus tendon	MEL + manual therapy + therapeutic exercise	Same manual therapy + therapeutic exercise	Yes	350 µA × 90 s = 31.5 mC/session	5; 1/week	Pain intensity (NPRS, 0–10)	6 months	Meta-analysis	25	1.50	1.80	25	4.10	3.40	MD	-2.60	-4.11 to -1.09	Calculated from group-level data used in the review synthesis	Original RCT reported between-group difference in change = −2.7 (95% CI −4.2 to −1.2).	EG: 6/25 muscle soreness; no serious treatment-related events.	No funding received
**Góngora-Rodríguez et al. (2025)**	Spain	Adults 18–65 years with supraspinatus tendinopathy; pain at tendon insertion/resisted movement/palpation;	MEL (EPTE®); supraspinatus tendon	MEL + PENS + eccentric therapeutic exercise	TENS + therapeutic ultrasound + same eccentric exercise	Yes	350 µA × 72 s = 25.2 mC/session	4; 1/week	Pain intensity (NPRS, 0–10)	24 weeks	Meta-analysis	23	0.60	0.90	23	3.60	2.10	MD	-3.00	-3.93 to -2.07	Calculated from group-level data used in the review	Original RCT reported between-group change score = −3.61; 95% CI	No adverse events reported.	No funding received

		positive Neer, Jobe, Hawkins–Kennedy.																			synthesis	not reported.		
**Arias-Buría et al. (2015)**	Spain	Chronic unilateral subacromial pain syndrome (≥3 months), NPRS ≥4/10, positive impingement signs, MRI-confirmed supraspinatus tendinopathy.	MEL (EPTE®); supraspinatus tendon	MEL + eccentric therapeutic exercise	Eccentric therapeutic exercise	Yes	350 µA × 72 s = 25.2 mC/session	4; 1/week	Disability (DASH, 0–100)	Post-treatment	Meta-analysis	17	11.10	8.80	19	20.80	7.40	Hedges g	-1.17	-1.87 to -0.47	Calculated from group-level data used in the review synthesis	Original RCT reported between-group difference in change = 9.5 (95% CI 1.9 to 17.2).	EG: 6/17 local post-treatment soreness; DOMS also reported; no serious treatment-related events.	Not reported
**de Miguel Valtierra et al. (2018)**	Spain	Unilateral shoulder pain ≥3 months; NPRS ≥4/10 during arm elevation; positive painful arc and ≥1 positive impingement/rotator cuff test.	MEL (EPTE®); supraspinatus tendon	MEL + manual therapy + therapeutic exercise	Same manual therapy + therapeutic exercise	Yes	350 µA × 90 s = 31.5 mC/session	5; 1/week	Disability (SPADI, 0–100)	Post-treatment	Meta-analysis	25	23.40	11.20	25	36.10	17.20	Hedges g	-0.86	-1.43 to -0.29	Calculated from group-level data used in the review synthesis	Original RCT reported between-group difference in change = −12.5 (95% CI −18.9 to −6.1).	EG: 6/25 muscle soreness; no serious treatment-related events.	No funding received
**Góngora-Rodríguez et al. (2024)**	Spain	Adults with symptomatic supraspinatus tendinopathy; ultrasound-confirmed tendon changes; pain with resisted movement; positive Jobe,	MEL (EPTE®); supraspinatus tendon	MEL + PENS + eccentric therapeutic exercise	TENS + therapeutic ultrasound + same eccentric exercise	Yes	350 µA × 72 s = 25.2 mC/session	4; 1/week	Disability (DASH, 0–100)	Post-treatment	Meta-analysis	25	14.00	15.90	25	31.80	19.90	Hedges g	-0.97	-1.55 to -0.39	Calculated from group-level data used in the review synthesis	Original RCT reported between-group change score = −33.16; 95% CI not	No adverse events reported.	No funding received

		Neer, Hawkins–Kennedy.																				reported.		
**Góngora-Rodríguez et al. (2025)**	Spain	Adults 18–65 years with supraspinatus tendinopathy; pain at tendon insertion/resisted movement/palpation; positive Neer, Jobe, Hawkins–Kennedy.	MEL (EPTE®); supraspinatus tendon	MEL + PENS + eccentric therapeutic exercise	TENS + therapeutic ultrasound + same eccentric exercise	Yes	350 µA × 72 s = 25.2 mC/session	4; 1/week	Disability (DASH, 0–100)	Post-treatment	Meta-analysis	23	11.30	10.10	23	33.50	24.10	Hedges g	-1.18	-1.80 to -0.56	Calculated from group-level data used in the review synthesis	Original RCT reported between-group change score = −36.83; 95% CI not reported.	No adverse events reported.	No funding received
**Magalhães et al. (2025)**	Brazil	Adults with chronic rotator cuff tendinopathy and shoulder pain ≥3 months.	MEL (MEP/IPM); rotator cuff tendon insertion region	MEL + therapeutic exercise	Same therapeutic exercise program	No	900 µA × 40 s = 36 mC/session	6; 1/week	Disability (SPADI, 0–100)	8 weeks	Meta-analysis	21	36.80	26.10	21	29.90	26.50	Hedges g	0.26	-0.34 to 0.85	Calculated from group-level data used in the review synthesis	Original RCT reported mixed-model between-group difference = 7.17 (95% CI −7.02 to 21.35).	EG: 12/21 nonserious events; CG: 14/21 nonserious expected events; no unexpected serious adverse effects.	No funding received
**Kużdżał et al. (2026)**	Poland	Imaging-confirmed subscapularis tendinopathy; age 25–65 years; shoulder pain ≥1 month; VAS ≥4/10; positive	EL (PNE/EPI); subscapularis tendon	EL + ESWT + manual therapy + exercise	Same ESWT + manual therapy + exercise	Yes	1.5 mA × 180 s × 3 = 810 mC/session	8; 1/week	Disability (DASH)	8 weeks	Meta-analysis	15	20.00	3.70	15	23.70	3.70	Hedges g	-0.97	-1.71 to -0.23	Calculated from group-level data used in the review	Original RCT reported between-group difference in change = −3.91	No treatment-related adverse events reported.	No funding received

		subscapularis test.																			synthesis	(95% CI −5.84 to −1.97).		
**de Miguel Valtierra et al. (2018)**	Spain	Unilateral shoulder pain ≥3 months; NPRS ≥4/10 during arm elevation; positive painful arc and ≥1 positive impingement/rotator cuff test.	MEL (EPTE®); supraspinatus tendon	MEL + manual therapy + therapeutic exercise	Same manual therapy + therapeutic exercise	Yes	350 µA × 90 s = 31.5 mC/session	5; 1/week	Disability (SPADI, 0–100)	3 months	Meta-analysis	25	13.50	8.90	25	30.60	17.60	MD	-17.10	-24.83 to -9.37	Calculated from group-level data used in the review synthesis	Original RCT reported between-group difference in change = −16.9 (95% CI −25.2 to −8.6).	EG: 6/25 muscle soreness; no serious treatment-related events.	No funding received
**Góngora-Rodríguez et al. (2025)**	Spain	Adults 18–65 years with supraspinatus tendinopathy; pain at tendon insertion/resisted movement/palpation; positive Neer, Jobe, Hawkins–Kennedy.	MEL (EPTE®); supraspinatus tendon	MEL + PENS + eccentric therapeutic exercise	TENS + therapeutic ultrasound + same eccentric exercise	Yes	350 µA × 72 s = 25.2 mC/session	4; 1/week	Disability (SPADI, 0–100)	12 weeks	Meta-analysis	23	7.00	7.30	23	42.20	22.30	MD	-35.20	-44.79 to -25.61	Calculated from group-level data used in the review synthesis	Original RCT reported between-group change score = −38.35; 95% CI not reported.	No adverse events reported.	No funding received
**de Miguel Valtierra et al. (2018)**	Spain	Unilateral shoulder pain ≥3 months; NPRS ≥4/10 during arm elevation; positive painful arc and ≥1 positive impingement	MEL (EPTE®); supraspinatus tendon	MEL + manual therapy + therapeutic exercise	Same manual therapy + therapeutic exercise	Yes	350 µA × 90 s = 31.5 mC/session	5; 1/week	Disability (SPADI, 0–100)	6 months	Meta-analysis	25	10.10	6.50	25	27.60	17.10	MD	-17.50	-24.67 to -10.33	Calculated from group-level data used in the review synthesis	Original RCT reported between-group difference in change = −17.3 (95% CI	EG: 6/25 muscle soreness; no serious treatment-related events.	No funding received

		/rotator cuff test.																				−25.5 to −9.1).		
**Góngora-Rodríguez et al. (2025)**	Spain	Adults 18–65 years with supraspinatus tendinopathy; pain at tendon insertion/resisted movement/palpation; positive Neer, Jobe, Hawkins–Kennedy.	MEL (EPTE®); supraspinatus tendon	MEL + PENS + eccentric therapeutic exercise	TENS + therapeutic ultrasound + same eccentric exercise	Yes	350 µA × 72 s = 25.2 mC/session	4; 1/week	Disability (SPADI, 0–100)	24 weeks	Meta-analysis	23	4.50	5.80	23	41.00	22.00	MD	-36.50	-45.80 to -27.20	Calculated from group-level data used in the review synthesis	Original RCT reported between-group change score = −39.61; 95% CI not reported.	No adverse events reported.	No funding received
**Kazemi et al. (2016)**	Spain	Infraspinatus trigger points and tendon pain ≥3 months; VAS ≥65/100; pain-limited abduction, internal rotation, and external rotation.	EL (EPI®); infraspinatus trigger points, tendon, or both	Three EL groups: trigger points; tendon; trigger points + tendon	No treatment (shared control)	Yes	6 mA; 4 s/application × 3 = 72 mC/session	3; 1/week	Shoulder abduction ROM (°)	Post-treatment	Meta-analysis	10	158.90	2.10	3	135.00	3.30	MD	23.90	19.95 to 27.85	Calculated from group-level data used in the review synthesis	EL – trigger points vs no treatment. Source reported restriction; converted to ROM as 180° − restriction. Shared control original n=10 split 3/3/4.	No harms or unintended effects reported.	Not reported
**Kazemi et al. (2016)**	Spain	Infraspinatus trigger points and tendon pain ≥3 months; VAS ≥65/100;	EL (EPI®); infraspinatus trigger points,	Three EL groups: trigger points; tendon;	No treatment (shared control)	Yes	6 mA; 4 s/application × 3 = 72 mC/session	3; 1/week	Shoulder abduction ROM (°)	Post-treatment	Meta-analysis	10	158.00	2.60	3	135.00	3.30	MD	23.00	18.93 to 27.07	Calculated from group-level data	EL – tendon vs no treatment. Source	No harms or unintended effects	Not reported

		pain-limited abduction, internal rotation, and external rotation.	tendon, or both	trigger points + tendon																	used in the review synthesis	reported restriction; converted to ROM as 180° – restriction. Shared control original n=10 split 3/3/4.	reported.	
**Kazemi et al. (2016)**	Spain	Infraspinatus trigger points and tendon pain ≥3 months; VAS ≥65/100; pain-limited abduction, internal rotation, and external rotation.	EL (EPI®); infraspinatus trigger points, tendon, or both	Three EL groups: trigger points; tendon; trigger points + tendon	No treatment (shared control)	Yes	6 mA; 4 s/application × 3 = 72 mC/session	3; 1/week	Shoulder abduction ROM (°)	Post-treatment	Meta-analysis	10	178.00	2.60	4	135.00	3.30	MD	43.00	39.39 to 46.61	Calculated from group-level data used in the review synthesis	EL – trigger points + tendon vs no treatment. Source reported restriction; converted to ROM as 180° – restriction. Shared control original n=10 split 3/3/4.	No harms or unintended effects reported.	Not reported
**Rodríguez-Huguet et al. (2020)**	Spain	Ultrasound-confirmed supraspinatus tendinopathy; age 25–60	MEL (EPTE®); supraspinatus tendon	MEL + eccentric therapeutic exercise	Trigger-point dry needling + same	Yes	350 µA × 72 s = 25.2 mC/session	4; 1/week	Shoulder abduction ROM (°)	Post-treatment	Meta-analysis	18	138.20	17.70	18	124.80	18.40	MD	13.40	1.61 to 25.19	Calculated from group-level data		No adverse events reported.	No funding received

		years; positive Hawkins–Kennedy and painful arc; active pain and tendon hyperalgesia.			eccentric exercise																used in the review synthesis			
**Góngora-Rodríguez et al. (2025)**	Spain	Adults 18–65 years with supraspinatus tendinopathy; pain at tendon insertion/resisted movement/palpation; positive Neer, Jobe, Hawkins–Kennedy.	MEL (EPTE®); supraspinatus tendon	MEL + PENS + eccentric therapeutic exercise	TENS + therapeutic ultrasound + same eccentric exercise	Yes	350 µA × 72 s = 25.2 mC/session	4; 1/week	Shoulder abduction ROM (°)	Post-treatment	Meta-analysis	23	143.40	17.50	23	120.00	23.40	MD	23.40	11.46 to 35.34	Calculated from group-level data used in the review synthesis	Original RCT reported between-group change score = 14.26°; 95% CI not reported.	No adverse events reported.	No funding received
**Kazemi et al. (2016)**	Spain	Infraspinatus trigger points and tendon pain ≥3 months; VAS ≥65/100; pain-limited abduction, internal rotation, and external rotation.	EL (EPI®); infraspinatus trigger points, tendon, or both	Three EL groups: trigger points; tendon; trigger points + tendon	No treatment (shared control)	Yes	6 mA; 4 s/application × 3 = 72 mC/session	3; 1/week	Shoulder internal rotation ROM (°)	Post-treatment	Meta-analysis	10	62.50	2.60	3	47.00	15.60	MD	15.50	-2.23 to 33.23	Calculated from group-level data used in the review synthesis	EL – trigger points vs no treatment. Source reported restriction; converted to ROM as 90° – restriction. Shared control original n=10 split 3/3/4.	No harms or unintended effects reported.	Not reported

**Kazemi et al. (2016)**	Spain	Infraspinatus trigger points and tendon pain ≥3 months; VAS ≥65/100; pain-limited abduction, internal rotation, and external rotation.	EL (EPI®); infraspinatus trigger points, tendon, or both	Three EL groups: trigger points; tendon; trigger points + tendon	No treatment (shared control)	Yes	6 mA; 4 s/application × 3 = 72 mC/session	3; 1/week	Shoulder internal rotation ROM (°)	Post-treatment	Meta-analysis	10	62.00	3.50	3	47.00	15.60	MD	15.00	-2.79 to 32.79	Calculated from group-level data used in the review synthesis	EL – tendon vs no treatment. Source reported restriction; converted to ROM as 90° – restriction. Shared control original n=10 split 3/3/4.	No harms or unintended effects reported.	Not reported
**Kazemi et al. (2016)**	Spain	Infraspinatus trigger points and tendon pain ≥3 months; VAS ≥65/100; pain-limited abduction, internal rotation, and external rotation.	EL (EPI®); infraspinatus trigger points, tendon, or both	Three EL groups: trigger points; tendon; trigger points + tendon	No treatment (shared control)	Yes	6 mA; 4 s/application × 3 = 72 mC/session	3; 1/week	Shoulder internal rotation ROM (°)	Post-treatment	Meta-analysis	10	87.00	4.20	4	47.00	15.60	MD	40.00	24.49 to 55.51	Calculated from group-level data used in the review synthesis	EL – trigger points + tendon vs no treatment. Source reported restriction; converted to ROM as 90° – restriction. Shared control original n=10 split 3/3/4.	No harms or unintended effects reported.	Not reported

**Rodríguez-Huguet et al. (2020)**	Spain	Ultrasound-confirmed supraspinatus tendinopathy; age 25–60 years; positive Hawkins–Kennedy and painful arc; active pain and tendon hyperalgesia.	MEL (EPTE®); supraspinatus tendon	MEL + eccentric therapeutic exercise	Trigger-point dry needling + same eccentric exercise	Yes	350 µA × 72 s = 25.2 mC/session	4; 1/week	Shoulder internal rotation ROM (°)	Post-treatment	Meta-analysis	18	86.90	9.70	18	69.90	11.00	MD	17.00	10.22 to 23.78	Calculated from group-level data used in the review synthesis		No adverse events reported.	No funding received
**Góngora-Rodríguez et al. (2025)**	Spain	Adults 18–65 years with supraspinatus tendinopathy; pain at tendon insertion/resisted movement/palpation; positive Neer, Jobe, Hawkins–Kennedy.	MEL (EPTE®); supraspinatus tendon	MEL + PENS + eccentric therapeutic exercise	TENS + therapeutic ultrasound + same eccentric exercise	Yes	350 µA × 72 s = 25.2 mC/session	4; 1/week	Shoulder internal rotation ROM (°)	Post-treatment	Meta-analysis	23	85.70	8.00	23	67.10	15.20	MD	18.60	11.58 to 25.62	Calculated from group-level data used in the review synthesis	Original RCT reported between-group change score = 16.83°; 95% CI not reported.	No adverse events reported.	No funding received
**Kużdżał et al. (2026)**	Poland	Imaging-confirmed subscapularis tendinopathy; age 25–65 years; shoulder pain ≥1 month; VAS ≥4/10; positive subscapularis test.	EL (PNE/EPI); subscapularis tendon	EL + ESWT + manual therapy + exercise	Same ESWT + manual therapy + exercise	Yes	1.5 mA × 180 s × 3 = 810 mC/session	8; 1/week	Shoulder internal rotation ROM (°)	8 weeks	Meta-analysis	15	77.10	6.50	15	72.20	4.20	MD	4.90	0.98 to 8.82	Calculated from group-level data used in the review synthesis	Original RCT reported between-group difference in change = 5.29° (95% CI 4.18 to 6.39).	No treatment-related adverse events reported.	No funding received
**Rodríguez-Huguet et al. (2020)**	Spain	Ultrasound-confirmed supraspinatus tendinopathy	MEL (EPTE®); supraspi	MEL + eccentric therape	Trigger-point dry needling + same	Yes	350 µA × 72 s = 25.2 mC/session	4; 1/week	Shoulder flexion ROM (°)	Post-treatment	Meta-analysis	18	152.20	12.60	18	143.90	20.60	MD	8.30	-2.86 to 19.46	Calculated from group-level	Original RCT reported adjuste	No adverse events	No funding received

		; age 25–60 years; positive Hawkins–Kennedy and painful arc; active pain and tendon hyperalgesia.	natus tendon	utic exercise	eccentric exercise																data used in the review synthesis	d between-group difference = 10.49° (95% CI −1.98 to 22.95).	reported.	
**Góngora-Rodríguez et al. (2025)**	Spain	Adults 18–65 years with supraspinatus tendinopathy; pain at tendon insertion/resisted movement/palpation; positive Neer, Jobe, Hawkins–Kennedy.	MEL (EPTE®); supraspinatus tendon	MEL + PENS + eccentric therapeutic exercise	TENS + therapeutic ultrasound + same eccentric exercise	Yes	350 μA × 72 s = 25.2 mC/session	4; 1/week	Shoulder flexion ROM (°)	Post-treatment	Meta-analysis	23	155.00	12.70	23	138.40	23.70	MD	16.60	5.61 to 27.59	Calculated from group-level data used in the review synthesis	Original RCT reported between-group change score = 22.70°; 95% CI not reported.	No adverse events reported.	No funding received
**Kazemi et al. (2016)**	Spain	Infraspinatus trigger points and tendon pain ≥3 months; VAS ≥65/100; pain-limited abduction, internal rotation, and external rotation.	EL (EPI®); infraspinatus trigger points, tendon, or both	Three EL groups: trigger points; tendon; trigger points + tendon	No treatment (shared control)	Yes	6 mA; 4 s/application × 3 = 72 mC/session	3; 1/week	External rotation restriction (°)	Post-treatment	Narrative only	10	29.00	3.94	10	50.00	4.08	MD	-21.00	-24.52 to -17.48	Calculated from group-level data used in the review synthesis	EL – trigger points vs no treatment. Restriction outcome retained as reported; lower values indicate less residual restriction. 0 ±	No harms or unintended effects reported.	Not reported

																						0 represents no residual restriction.		
**Kazemi et al. (2016)**	Spain	Infraspinatus trigger points and tendon pain ≥3 months; VAS ≥65/100; pain-limited abduction, internal rotation, and external rotation.	EL (EPI®); infraspinatus trigger points, tendon, or both	Three EL groups: trigger points; tendon; trigger points + tendon	No treatment (shared control)	Yes	6 mA; 4 s/application × 3 = 72 mC/session	3; 1/week	External rotation restriction (°)	Post-treatment	Narrative only	10	29.50	4.38	10	50.00	4.08	MD	-20.50	-24.21 to -16.79	Calculated from group-level data used in the review synthesis	EL – tendon vs no treatment. Restriction outcome retained as reported; lower values indicate less residual restriction. 0 ± 0 represents no residual restriction.	No harms or unintended effects reported.	Not reported
**Kazemi et al. (2016)**	Spain	Infraspinatus trigger points and tendon pain ≥3 months; VAS ≥65/100; pain-limited abduction, internal rotation, and external rotation.	EL (EPI®); infraspinatus trigger points, tendon, or both	Three EL groups: trigger points; tendon; trigger points + tendon	No treatment (shared control)	Yes	6 mA; 4 s/application × 3 = 72 mC/session	3; 1/week	External rotation restriction (°)	Post-treatment	Narrative only	10	0.00	0.00	10	50.00	4.08	MD	-50.00	-52.53 to -47.47	Calculated from group-level data used in the review synthesis	EL – trigger points + tendon vs no treatment. Restriction outcome retained as	No harms or unintended effects reported.	Not reported

																						reported; lower values indicate less residual restriction. 0 ± 0 represents no residual restriction.		
**Rodríguez-Huguet et al. (2020)**	Spain	Ultrasound-confirmed supraspinatus tendinopathy; age 25–60 years; positive Hawkins–Kennedy and painful arc; active pain and tendon hyperalgesia.	MEL (EPTE®); supraspinatus tendon	MEL + eccentric therapeutic exercise	Trigger-point dry needling + same eccentric exercise	Yes	350 µA × 72 s = 25.2 mC/session	4; 1/week	Shoulder external rotation ROM (°)	Post-treatment	Narrative only	18	86.83	5.88	18	70.33	12.27	MD	16.50	10.21 to 22.79	Calculated from group-level data used in the review synthesis	Original RCT reported adjusted between-group difference = 19.27° (95% CI 12.64 to 25.90).	No adverse events reported.	No funding received
**Góngora-Rodríguez et al. (2025)**	Spain	Adults 18–65 years with supraspinatus tendinopathy; pain at tendon insertion/resisted movement/palpation; positive Neer, Jobe, Hawkins–Kennedy.	MEL (EPTE®); supraspinatus tendon	MEL + PENS + eccentric therapeutic exercise	TENS + therapeutic ultrasound + same eccentric exercise	Yes	350 µA × 72 s = 25.2 mC/session	4; 1/week	Shoulder external rotation ROM (°)	Post-treatment	Narrative only	23	84.04	10.77	23	70.96	17.14	MD	13.08	4.81 to 21.35	Calculated from group-level data used in the review synthesis	Original RCT reported between-group change score = 12.70°; 95% CI not reported.	No adverse events reported.	No funding received

**de Miguel Valtierra et al. (2018)**	Spain	Unilateral shoulder pain ≥3 months; NPRS ≥4/10 during arm elevation; positive painful arc and ≥1 positive impingement/rotator cuff test.	MEL (EPTE®); supraspinatus tendon	MEL + manual therapy + therapeutic exercise	Same manual therapy + therapeutic exercise	Yes	350 µA × 90 s = 31.5 mC/session	5; 1/week	PPT – deltoid (kg/cm²)	Post-treatment	Narrative only	25	7.00	2.30	25	7.10	2.60	MD	-0.10	-1.46 to 1.26	Calculated from group-level data used in the review synthesis	Original RCT reported between-group difference in change = 0.1 (95% CI −0.7 to 0.9).	EG: 6/25 muscle soreness; no serious treatment-related events.	No funding received
**Rodríguez-Huguet et al. (2020)**	Spain	Ultrasound-confirmed supraspinatus tendinopathy; age 25–60 years; positive Hawkins–Kennedy and painful arc; active pain and tendon hyperalgesia.	MEL (EPTE®); supraspinatus tendon	MEL + eccentric therapeutic exercise	Trigger-point dry needling + same eccentric exercise	Yes	350 µA × 72 s = 25.2 mC/session	4; 1/week	PPT – distal supraspinatus (kg/cm²)	Post-treatment	Narrative only	18	3.90	1.09	18	3.03	0.75	MD	0.87	0.26 to 1.48	Calculated from group-level data used in the review synthesis	Original RCT reported adjusted between-group difference = 1.00 (95% CI 0.32 to 1.68).	No adverse events reported.	No funding received
**Góngora-Rodríguez et al. (2025)**	Spain	Adults 18–65 years with supraspinatus tendinopathy; pain at tendon insertion/resisted movement/palpation; positive Neer, Jobe, Hawkins–Kennedy.	MEL (EPTE®); supraspinatus tendon	MEL + PENS + eccentric therapeutic exercise	TENS + therapeutic ultrasound + same eccentric exercise	Yes	350 µA × 72 s = 25.2 mC/session	4; 1/week	PPT – distal supraspinatus	Post-treatment	Narrative only	23	4.06	1.03	23	3.20	0.90	MD	0.86	0.30 to 1.42	Calculated from group-level data used in the review synthesis	Original RCT reported between-group change score = 0.89; 95% CI not reported.	No adverse events reported.	No funding received
**Kużdżał et al. (2026)**	Poland	Imaging-confirmed subscapularis tendinopathy	EL (PNE/EPI); subscap	EL + ESWT + manual therapy	Same ESWT + manual therapy	Yes	1.5 mA × 180 s × 3 = 810	8; 1/week	PPT – distal subscap	8 weeks	Narrative only	15	93.58	3.51	15	83.59	2.79	MD	9.99	7.72 to 12.26	Calculated from group	Original RCT reported	No treatment-related	No funding

	; age 25–65 years; shoulder pain ≥1 month; VAS ≥4/10; positive subscapularis test.	ularis tendon	+ exercise	+ exercise	mC/session	ularis (kPa)		-level data used in the review synthesis	between-group difference in change = 10.01 kPa (95% CI 9.07 to 10.94).	adverse events reported.	received

Abbreviations: CG, control group; CI, confidence interval; DASH, Disabilities of the Arm, Shoulder and Hand; DOMS, delayed-onset muscle soreness; EG, experimental group; EL, electrolysis; EPI®, intratissue percutaneous electrolysis; EPTE®, therapeutic percutaneous electrolysis; ESWT, extracorporeal shockwave therapy; g, Hedges' g; MD, mean difference; MEL, microelectrolysis; MRI, magnetic resonance imaging; NPRS, Numeric Pain Rating Scale; PEDro, Physiotherapy Evidence Database; PNE, percutaneous needle electrolysis; PENS, percutaneous electrical nerve stimulation; PPT, pressure pain threshold; ROM, range of motion; SD, standard deviation; SPADI, Shoulder Pain and Disability Index; TE, therapeutic exercise; TENS, transcutaneous electrical nerve stimulation; US, therapeutic ultrasound; VAS, Visual Analogue Scale.

Supplementary Table S3. Diagnostic and Eligibility Criteria of the Included RCTs.

First author (year)	Diagnosis	Selection criteria
Arias-Buría (2015) [37]	SAPS with supraspinatus tendinopathy	Inclusion: Chronic unilateral SAPS (≥3 months), NPRS ≥4/10, positive impingement signs, and MRI-confirmed supraspinatus tendinopathy.<br><br>Exclusion: Bilateral symptoms, age <18 or >65 years, fracture/dislocation, cervical radiculopathy, corticosteroid injection, fibromyalgia, previous shoulder/neck surgery, or recent neck–shoulder treatment.
Kazemi (2016) [38]	Symptomatic infraspinatus tendon involvement	Inclusion: Infraspinatus MTrPs, infraspinatus tendon pain ≥3 months, VAS ≥65/100, and pain-limited shoulder abduction, internal rotation, and external rotation.<br><br>Exclusion: Previous surgery or systemic disease.
de Miguel Valtierra (2018) [39]	SAPS with supraspinatus tendon involvement	Inclusion: Unilateral shoulder pain ≥3 months, NPRS ≥4/10 during arm elevation, positive painful arc test, and ≥1 positive impingement/rotator cuff test (Hawkins–Kennedy, Neer, Empty Can, Drop Arm, or Lift-Off).<br><br>Exclusion: Bilateral shoulder symptoms, age <18 or >65 years, history of shoulder fracture, cervical radiculopathy, previous corticosteroid injection, fibromyalgia, previous shoulder or neck surgery, or neck–shoulder treatment during the previous year.
Rodríguez-Huguet (2020) [40]	Supraspinatus tendinopathy	Inclusion: USG-confirmed supraspinatus tendinopathy, age 25–60 years, positive Hawkins–Kennedy and painful arc tests, active pain, and tendon hyperalgesia.<br><br>Exclusion: Previous arm surgery, fracture/dislocation, recent severe disorder, bilateral symptoms, cervical radiculopathy, fibromyalgia, pregnancy, pacemaker, cancer, active infection, or generalized lymphedema.
Góngora-Rodríguez (2024) [41]	Supraspinatus tendinopathy	Inclusion: Age 18–65 years, symptomatic supraspinatus tendinopathy, pain at the supraspinatus insertion, ultrasound-confirmed structural tendon changes, pain during resisted shoulder movements, and positive Jobe, Neer, and Hawkins–Kennedy tests.<br><br>Exclusion: Bilateral involvement, previous electrolysis or PENS treatment, recent severe trauma, shoulder surgery, previous fracture/dislocation, regular medication use, pregnancy, fibromyalgia, pacemaker, cancer, infection, or lymphedema.
Góngora-Rodríguez (2025) [42]	Supraspinatus tendinopathy	Inclusion: Adults aged 18–65 years with supraspinatus tendinopathy, pain at the tendon insertion, pain during resisted movement and palpation, positive Neer, Jobe, and Hawkins–Kennedy tests, no signs of nerve irritation, and no improvement with drug therapy.

		Exclusion: Shoulder pain due to trauma, fractures, neoplasia, severe osteoporosis, infectious or inflammatory disorders, pacemaker, congenital anomalies, pregnancy, or previous microelectrolysis or PENS treatment.
Magalhães (2025) [43]	Chronic imaging-confirmed RC tendinopathy	Inclusion: Chronic RC tendinopathy confirmed by MRI/USG, age ≥18 years, and shoulder pain ≥3 months.<br><br>Exclusion: Contraindications to MEP®, rheumatologic/systemic disease, previous shoulder surgery, recent corticosteroid injection, adhesive capsulitis, labral injury, or shoulder fracture.
Kużdżał (2026) [44]	Subscapularis tendinopathy	Inclusion: USG/MRI-confirmed subscapularis tendinopathy, age 25–65 years, shoulder pain ≥1 month, VAS ≥4/10, positive subscapularis test, and reduced internal rotation strength.<br><br>Exclusion: Previous shoulder surgery/injection, recent NSAID use, rotator cuff tear, SAIS, AC joint degeneration, tendon calcification, systemic musculoskeletal disease, pregnancy, or recent trial participation.

Abbreviations: AC, acromioclavicular; MRI, magnetic resonance imaging; MTrPs, myofascial trigger points; NPRS, Numeric Pain Rating Scale; NSAIDs, nonsteroidal anti-inflammatory drugs; PENS, percutaneous electrical nerve stimulation; RC, rotator cuff; SAIS, subacromial impingement syndrome; SAPS, subacromial pain syndrome; USG, ultrasound imaging; VAS, visual analogue scale.

Supplementary Table S4. Methodological quality of included studies assessed using the PEDro scale.

Author (year) Country	PEDro scale criteria											
	**Criteria 1***	**Criteria 2**	**Criteria 3**	**Criteria 4**	**Criteria 5**	**Criteria 6**	**Criteria 7**	**Criteria 8**	**Criteria 9**	**Criteria 10**	**Criteria 11**	**PEDro score**
Arias-Buría (2015)** Spain	Yes	Yes	Yes	Yes	No	No	Yes	Yes	No	Yes	Yes	7
Kazemi (2016)+ Spain	Yes	Yes	No	No	Yes	No	No	Yes	No	Yes	Yes	5
de Miguel Valtierra (2018)+ Spain	Yes	Yes	Yes	Yes	Yes	No	Yes	Yes	Yes	Yes	Yes	9
Rodríguez-Huguet (2020)+ Spain	Yes	Yes	Yes	Yes	No	No	Yes	Yes	No	Yes	Yes	7
Góngora-Rodríguez (2024)+ Spain	Yes	Yes	No	No	No	No	Yes	Yes	No	Yes	Yes	5
Góngora-Rodríguez (2025)** Spain	Yes	Yes	Yes	No	No	Yes	Yes	Yes	No	Yes	Yes	7
Magalhães (2025)+ Brazil	Yes	Yes	Yes	No	No	Yes	Yes	Yes	Yes	Yes	Yes	8
Kuźdżał (2026)+ Poland	Yes	Yes	Yes	No	No	Yes	Yes	Yes	Yes	Yes	Yes	8

PEDro (Physiotherapy Evidence Database) scale criteria:

(1) Eligibility criteria were specified; (2) Random allocation; (3) Concealed allocation; (4) Baseline comparability; (5) Blind subjects; (6) Blind therapists; (7) Blind assessors; (8) Adequate follow-up; (9) Intention-to-treat analysis; (10) Between-group comparisons; (11) Point estimates and variability

*Eligibility criteria item does not contribute to total score

**Score confirmed in PEDro database

+Score determined by researchers (Not available in PEDro database)
